# Exploring *murE* protein inhibitors of *Tropheryma whipplei* through pharmacoinformatic approaches incorporating solubility-enhancing formulation insights

**DOI:** 10.3389/fphar.2025.1630038

**Published:** 2025-08-14

**Authors:** Zarrin Basharat, Calvin R. Wei, Madiha Islam, Ibrar Ahmed, Hanan A. Ogaly, Fatimah A. M. Al-Zahrani, Yasir Waheed, Seil Kim

**Affiliations:** ^1^ Alpha Genomics Private Limited, Islamabad, Pakistan; ^2^ Department of Research and Development, Shing Huei Group, Taipei, Taiwan; ^3^ Department of Biotechnology and Genetic Engineering, Hazara University, Mansehra, Pakistan; ^4^ Microbiological Analysis Team, Group of Biometrology, The Korea Research Institute of Standards and Science (KRISS), Daejeon, Republic of Korea; ^5^ Chemistry Department, College of Science, King Khalid University, Abha, Saudi Arabia; ^6^ NUST School of Health Sciences, National University of Sciences and Technology (NUST), Islamabad, Pakistan; ^7^ Operational Research Center in Healthcare, Near East University, Nicosia, Türkiye; ^8^ Széchenyi István University, Győr, Hungary; ^9^ VIZJA University, Warsaw, Poland; ^10^ Convergent Research Center for Emerging Virus Infection, Korea Research Institute of Chemical Technology, Daejeon, Republic of Korea; ^11^ Department of Bio-Analysis Science, University of Science and Technology, Daejeon, Republic of Korea

**Keywords:** Tropheryma whipplei, Whipple’s disease, Ayurveda, virtual screening, MD simulation

## Abstract

*Tropheryma whipplei* the causative agent of Whipple disease, presents a diagnostic challenge due to its diverse symptomatology, including weight loss, abdominal pain, diarrhea, joint pain, fever, and occasionally neurological manifestations. Its resistance to fluoroquinolones complicates treatment further. Traditional methods for antibiotic susceptibility testing are ineffective as *Tropheryma whipplei* cannot be cultured in axenic media. To address this, we explored potential drug targets within its core genome as no drug targets from this bacterium have been studied so far. *murE*, a macrolide-resistant enzyme, emerged as a promising candidate exhibiting both resistance and drug target characteristics. We screened over 1,000 lead-like Ayurvedic compounds against the target enzyme UDP-N-acetylmuramyl-tripeptide synthetase and identified three promising candidates: (1) Ergost-5-en-3-ol (3beta,24xi), (2) [6]-Gingerdiol 3-monoacetate, and (3) Valtrate. DiffDock and GNINA rescoring yielded consistent binding strength rankings. Molecular dynamics simulations over 100 nanoseconds confirmed stable interactions with these compounds. ADMET analysis indicated low water solubility, but coupling with cyclodextrin SBE-β-CD improved solubility. None of the compounds showed hepatotoxic effects, though Valtrate exhibited AMES toxicity. Based on the favorable properties, we propose scaffold hopping and further *in vitro/in vivo* studies on [6]-Gingerdiol 3-monoacetate. Our findings offer potential avenues for combating *T. whipplei* infections, addressing the limitations posed by antibiotic resistance.

## 1 Introduction

*Tropheryma whipplei* is a rod-shaped Gram-positive bacterium is a member of the Actinomycetales order and phylum Actinobacteria (Liu, 2024). It is usually found in drinking water, soil, and sewage (Marth, 2015) and is a contributing factor of endocarditis and Whipple’s disease (Marth et al., 2016). *Tropheryma whipplei* infection is more prevalent in sewage workers in western countries and in children under the age of seven in unhygienic countries (Keita et al., 2015). The brain, eyes, skin, joints, and heart are among the human organs that can be affected by the rare, yet persistent Whipple’s sickness. This disease manifests with diverse symptoms, often including diarrhea, weight loss, abdominal pain, fever, fatigue, joint pain, uveitis, skin problems, and neurological issues (Marth, 2015). Approximately 15% of infections by pathogens are asymptomatic. This bacterium is more likely to infect men in a higher proportion (73%–87%) and targets them between the ages of 48 and 54 (Ruffer et al., 2023). Although the mortality rate is still unknown, it may be lethal if not treated appropriately (Albekairi et al., 2022).

The oral-oral and oral-fecal pathways are the common routes by which *T. whipplei* spreads (Shams et al., 2021). Humans are the only organisms that harbor the infection as there is no known vector of these bacteria. It multiplies in intestinal mucosa macrophages and can persist after phagocytosis (Al Moussawi et al., 2010; Braubach et al., 2017). The bacteria can remain in a patient for a long time after they have acquired an immune response to the initial encounter, which allows it to propagate across the community (Moos et al., 2025). Compared to the stool sample, the saliva samples contain a much higher concentration of bacteria, indicating that it is a common gut bacterium (Dolmans et al., 2017).

Despite advancements in diagnostic procedures and understanding, diagnosing and treating *T. whipplei* infection remains challenging due to its complex symptoms and rarity. Diagnosis involves biopsy of the affected tissue, typically the small intestine (Anton-Vazquez et al., 2022). Nowadays, a number of techniques, such as polymerase chain reaction (PCR) and immunohistochemistry tests for different genes of interest on biopsy samples can also be used to provide a definitive diagnosis of Whipple’s disease (Baisden et al., 2002). The PCR assay has emerged as a crucial diagnostic tool for Whipple’s disease, particularly in cases where the diagnosis cannot be histologically confirmed or in individuals with peculiar presentations (Boulos et al., 2004; Melas et al., 2021).

Whipple’s disease can pose a lethal threat if left untreated. Research indicates a continuous prevalence of middle-aged males, late diagnosis, and increased risk of relapse with CNS symptoms (Hujoel et al., 2019). Since the majority of relapses occur in the central nervous system, which is also frequently involved in the disease, antibiotic medication ought to be initiated as soon as possible and to prevent neurological relapses, it is imperative to administer drugs with high absorption rates into the CNS (Renon et al., 2012; Bureš et al., 2013; Paddock et al., 2022). Trimethoprim/sulfamethoxazole, due to its ability to penetrate the blood-brain barrier, is often chosen for antibiotic treatment in cases where the CNS is involved (El-Abassi et al., 2023). Despite its efficacy, the use of such antibiotics can lead to the development of antibiotic resistance in patients. Additionally, treatment failure or relapse has been reported in patients undergoing trimethoprim/sulfamethoxazole therapy (García-Álvarez and Oteo, 2021). Treatment with doxycycline and hydroxychloroquine or ceftriaxone have also been reported (Foteinogiannopoulou et al., 2021; Portillo et al., 2024). The issues with antibiotic therapy include resistance and adverse reactions like Jarisch-Herxheimer reaction, particularly in individuals receiving immunosuppressive therapy or those with CNS diseases, presenting symptoms akin to systemic inflammatory response syndrome (Fong and Fong, 2020). Dysbiosis of gut microbiota caused by antibiotics can also have significant implications for Whipple’s disease patients due to the direct link between the disease and the intestine, as well as the gut-brain axis.

Considering these implications, exploring alternative treatment approaches like plant compounds becomes imperative. Previously, molecules from plants, including polyphenols, flavonoids, etc., have shown promise in modulating gut microbiota composition, reducing inflammation, and promoting gut health (Direito et al., 2021; Fabbrini et al., 2022; Açar et al., 2023). Plant-based drugs have long served as a line of defense in sustaining health and treating infections. Plant compounds exert their effects through various mechanisms, including antimicrobial activity against pathogens (Simonetti et al., 2020; Vaou et al., 2021), promotion of beneficial bacteria growth (Santhiravel et al., 2022), and modulation of immune responses (Alhazmi et al., 2021). Moreover, unlike antibiotics, plant compounds are less likely to induce antibiotic resistance and cause adverse effects such as dysbiosis (Zhang L. et al., 2022; Vignesh et al., 2023). By targeting specific pathways involved in disease pathogenesis while preserving the gut microbiota balance, plant-based therapies offer a potentially safer and more sustainable treatment option for Whipple’s disease patients. Hence, the primary aim of this research was to screen lead-like ayurvedic compounds to inhibit bacterial growth as use of drugs and antibiotics impose several effects.

## 2 Materials and methods

### 2.1 Data retrieval and pan-genomics

The available genomes of *T. whipplei* were acquired from BV-BRC database (Olson et al., 2023). The genome size and gene composition of each component was calculated. BPGA(Chaudhari et al., 2016) was used to analyze the pan-genome of *T. whipplei*. It facilitates the comparative analysis across multiple genomes of the same species using protein (.faa) or genbank format (.gbk) files. USEARCH clustering algorithm was used (70% similarity cut-off). The clustered output was used to generate a tab-delimited binary gene presence–absence matrix (pan-matrix), which served as the basis for iterative pan-genome profiling (19 iterations, equal to the number of genomes) and the core (genes found in every strains), dispensable (genes found in some strains), and the accessory genome (genes distinctive to individual strains) was identified (Basharat and Meshal, 2023). Functional enrichment analysis was done to identify significantly overrepresented gene categories or pathways within the pan-genome components (Sancho et al., 2022). This included annotating gene clusters with functional categories (e.g., COG, KEGG) and applying statistical tests to identify categories/pathways enriched in each genome component. The aim was to uncover functional diversity and adaptive traits across *T. whipplei* strains. This analysis offered insights into the functional diversity and potential adaptive traits of *T. whipplei* strains. Additionally, a phylogenetic tree was constructed based on orthologous gene clusters from the core and pan-genomes to infer evolutionary relationships among the strains. The neighbor-joining method was used, and the resulting tree was visualized and interpreted to assess genetic relatedness and evolutionary divergence within the species (Inglin et al., 2018).

### 2.2 Drug target identification and structure analysis

The core genome consisting of genes conserved across all strains was used for this purpose. Subtractive genomics was performed using installed BLAST to identify potential drug targets by comparing the genome of the target organism with the human genome, essential gene databases and beneficial microbiota of the gut (Uddin and Saeed, 2014; Khan et al., 2022). Aim was to identify genes present in the target organism but absent or significantly different in non-pathogenic counterparts. These unique or significantly different genes are potential drug targets specific to the pathogen (Gupta et al., 2017; Gupta et al., 2019). The final set of subtracted sequences were analyzed based significance of the protein role in pathogenicity or survival, and druggability. Among these, *murE* (coding for UDP-N-acetylmuramoyl-L-alanyl-D-glutamate--2,6-diaminopimelate ligase) was selected for downstream analysis (Kumar et al., 2023). This particular protein contributes to peptidoglycan biosynthesis, which is crucial for cell wall formation, shape and integrity maintenance, thereby influencing bacterial survival (Amera et al., 2019). MurE selectively incorporates meso-diaminopimelic acid (m-A2p.m.) in most Gram-negative bacteria and Bacilli, whereas Gram-positive bacteria typically use L-lysine (Hervin et al., 2023). Its function requires ATP to activate the carboxyl group of the peptide stem through acyl-phosphate formation, facilitating nucleophilic attack by the amino group of m-A2pm. This reaction results in the production of ADP and inorganic phosphate as byproducts. *murE* is often encoded in conserved operons, such as the dcw cluster, and can form chimeric complexes, like *murE-murF* in *Bordetella pertussis*, to enhance pathway efficiency (Gaur and Bera, 2022; Shirakawa et al., 2023). Active site of this enzyme enforces strict substrate specificity, ensuring proper amino acid incorporation and any errors in this process can lead to bacterial cell lysis. Notably, *murE* is absent in mammalian cells, making it an attractive and validated target for novel antibiotics (Gaur and Bera, 2022). Inhibitors that disrupt its activity could provide an effective strategy for combating drug-resistant pathogens (El Zoeiby et al., 2003). Consequently, targeting Mur enzymes for inhibition presents a promising avenue for the development of new antibacterial agents (Amera et al., 2020; Cazzaniga et al., 2021; Koatale et al., 2024). Structure of the selected target (UDP-N-acetylmuramyl-tripeptide synthetase, also known as MurE synthetase) was obtained from AlphaFold (Accession: AF-Q83HJ9-F1-v4) (Jumper et al., 2021). The metrics, such as per-residue model confidence score (pLDDT), and predicted aligned error (PAE) were evaluated to measure consistency with experimental data (Guo et al., 2022). This assessment helped to assess the reliability and accuracy of the predicted structures.

### 2.3 Virtual screening

A database of over 2,000 Ayurvedic compounds was compiled (Choudhary and Singh, 2022) and subsequently filtered using druggability and lead-like criteria, reducing the set to approximately 1,000 compounds. For these 1000 compounds, protonation states, tautomeric forms, and ionization states of the ligands were adjusted as required.

AutoDock Vina (Nguyen et al., 2019; Eberhardt et al., 2021) was used for molecular docking-based screening techniques to identify small molecules or compounds that could bind to the target protein with high affinity and specificity (Shahroz et al., 2022). AutoDock Vina employs a scoring function that considers both the shape complementarity and the binding affinity between the ligand and protein. A grid of 25Ǻ was generated along the X, Y and Z-axis, around the target protein to define the search space for molecular docking (Alliouche et al., 2024). This centroid encompassed the active site where ligands were likely to bind. For every ligand, several docking poses were produced to explore different binding orientations and conformations (Xue et al., 2022). The generated docking poses were scored based on the binding affinity between the ligand and the target protein. The scoring function in AutoDock Vina provided an estimation of the binding free energy or affinity. The docking poses were ranked based on their scores, with lower scores indicating stronger binding (Tanchuk et al., 2016). The scoring was validated using the DiffDock (Corso et al., 2022) and GNINA(McNutt et al., 2021). DiffDock, a diffusion model-based docking approach, optimizes the positioning and orientation of small molecules relative to the target protein and assigns a confidence score to each predicted binding pose. The DiffDock confidence score assesses the certainty of the ligand fit, where a higher score suggests a better fit, while a lower score indicates uncertainty. Specifically, a confidence score (C) greater than 0 signifies high confidence, a score between −1.5 and 0 denotes moderate confidence, and a score below −1.5 is considered low confidence. The applicability of these confidence intervals depends on the protein-ligand complex size, with adjustments required for large ligands, protein complexes, or unbound conformations. To compare binding across different ligands, the binding poses were also assessed using GNINA, which integrates Convolutional Neural Network (CNN) modules and the Vinardo scoring function. Vinardo evaluates the energy of the ligand-protein interaction, where a more negative score indicates better binding, while a higher score suggests weaker binding (Zhang et al., 2024). CNN predicts the accuracy with which the ligand fits into the binding site of the target protein (Zhao et al., 2020). A higher score signifies better docking potential. Additionally, the CNN affinity score measures the strength of the ligand-protein interaction, considering factors such as shape complementarity, chemical bonds (e.g., hydrogen bonds, ionic interactions, Van der Waals forces), hydrophobic interactions, electrostatic interactions, and flexibility.

Hit Dexter 3 (Stork et al., 2018) was used to identify molecule promiscuity and SwissTarget Prediction (Daina et al., 2019) was used to identify macromolecular targets in humans. Hit Dexter is trained on PubChem data and focus on generating readouts from purified proteins or peptide data, under target-based assay category. This assay is trained on data obtained by biochemical or biophysical methods to measure the binding affinity or enzymatic activity of compounds against isolated target proteins. Conversely, cell-based assay, utilize living cell data to generate readouts. These assays are trained on data to measure specific protein-compound interactions within cellular environments, often employing fluorescence-based techniques or other molecular probes. Additionally, an extended selection of cell-based assay data, encompassing tissue-based and organism-based assays was also employed. These assays provide broader insights into compound effects, including nonspecific interactions such as cytotoxicity or organism-level responses.

### 2.4 Absorption, distribution, metabolism, excretion and toxicity (ADMET)

ADMET profiling was done using pKCSM (URL:https://biosig.lab.uq.edu.au/pkcsm/; retrieved 30 March 2024) (Pires et al., 2015) and SwissADME (URL: http://www.swissadme.ch/; retrieved 30 March 2024) (Daina et al., 2017). pKCSM uses machine learning algorithms trained on experimental data to estimate key properties related to pharmacokinetics and toxicity. SwissADME employs a combination of rule-based and machine learning methods to estimate various ADMET properties (Kumar et al., 2021). Estimations for several key properties, including physicochemical descriptors, drug-likeness, lipophilicity, distribution, absorption, excretion, metabolism, and toxicity-related parameters were obtained. This was to identify any potential issues or concerns associated with ligands that could hinder their development into drugs (Wu et al., 2020). For molecules with low water solubility and consequently poor oral bioavailability, cyclodextrin complexation was evaluated as a strategy to enhance their solubility using Formulation AI (https://formulationai.computpharm.org/; accessed 10 January 2025). While other solubility-enhancing methods exist, cyclodextrins were specifically chosen in this study due to their safety, efficacy, ease of formulation, and established regulatory acceptance. Their ability to enhance solubility while maintaining drug stability and minimizing toxicity makes them a compelling option compared to lipid-based carriers, prodrugs, or nanocarriers. SMILE format of priority ligands was fed and temperature was kept 310K, while pH was kept 7. Eight cyclodextrins were evaluated, including α-cyclodextrin (α-CD), β-cyclodextrin (β-CD), γ-cyclodextrin (γ-CD), 2-hydroxypropyl-β-cyclodextrin (HP-β-CD), methyl-β-cyclodextrin (M-β-CD), hydroxypropyl-γ-cyclodextrin (HP-γ-CD), sulfobutylether-β-cyclodextrin (SBE-β-CD), and randomly methylated-β-cyclodextrin (RM-β-CD). These cyclodextrins differ in their cavity size, substitution patterns, and solubility-enhancing properties. Specifically, α-CD has a smaller cavity and is typically used for smaller molecules (Sandilya et al., 2020), β-CD is commonly studied and utilized, providing moderate solubility enhancement, while γ-CD has a larger cavity suitable for larger molecules (Zhang et al., 2007). The modified cyclodextrins such as HP-β-CD, M-β-CD, SBE-β-CD and RM-β-CD offer improved solubility and stability profiles compared to their native counterparts. The energy values of complexation were noted and analyzed further.

### 2.5 Molecular dynamics (MD) simulation

The GROMACS MD simulation package (https://www.gromacs.org/) (Abraham et al., 2015) version 2021 was utilized to investigate stability of prioritized complexes. Protein and ligand, were prepared by adding missing atoms and assigning OPLS-AA force field parameters to the biomolecules (Beckstein et al., 2014). The system was solvated with water TIP3P, and in order to balance the overall charge, counter ions were added. To relax the initial structure and alleviate steric clashes, an energy-minimization process was carried out. During the energy minimization, position restraints were applied to the biomolecules to prevent excessive perturbations. Subsequently, a stepwise equilibration process was carried out to adjust the system gradually to the desired conditions. This process involved solvent equilibration, temperature equilibration, and pressure equilibration steps. Position restraints were also applied during equilibration to maintain the bimolecular structure. Once equilibrated, the production run was initiated for data collection. MD simulations were performed for 100 ns. Trajectory data obtained from the production run were analyzed to extract valuable insights into the system. Structural properties such as secondary structure content, radius of gyration, and root mean square deviation (RMSD) were computed. Dynamic properties like hydrogen bonding and root mean square fluctuations (RMSF) were also computed. Time-resolved principal component analysis (PCA) was performed on hydrogen bond and RMSD data extracted from GROMACS. xvg files, specifically complex RMSD and total hydrogen bonds. Feature values were sampled at defined time intervals and compiled into a matrix, followed by Z-score standardization. PCA was conducted using the scikit-learn library, retaining the first two principal components (PC1 and PC2) to capture the major variance in structural dynamics over time. Complex-specific trajectories were visualized in PC space using matplotlib and seaborn, enabling interpretation of temporal conformational shifts. The resulting plots were used to assess whether the systems remained within a confined conformational space, indicating structural stability throughout the simulation.

### 2.6 Physiologically based pharmacokinetic (PBPK) profiling

PBPK profiling was conducted using the GastroPlus software v9.8.3 (Romero et al., 2020; Costa et al., 2024). The study focused on evaluating the pharmacokinetics of top compounds in three distinct categories of 50 people population each: normal, renally impaired, and cirrhotic. The anatomical and physiological parameters specific to the study group were incorporated into the models. These parameters included organ volumes, blood flow rates, tissue permeabilities, and enzyme activity levels (Alzamami et al., 2024). Molecular weight and lipophilicity of the compounds was also integrated. Once the models were established, simulations were performed to predict the pharmacokinetic behavior. The simulations took into account factors such as oral absorption, distribution within various tissues, and metabolism by specific enzymes, and elimination via renal and hepatic pathways (Miller et al., 2019; Soliman et al., 2022). A comparative analysis of drug exposure and disposition among the different groups was done.

## 3 Results

*Tropheryma whipplei* has a 46% GC content and is the sole pathogen currently identified with a short genome (927 kb) (Bentley et al., 2003). The bacterium shows little variability, with different strains of *T. whipplei* showing 99% similarity (Bulut et al., 2022).

### 3.1 Pan-genome analysis

**FIGURE 1:**
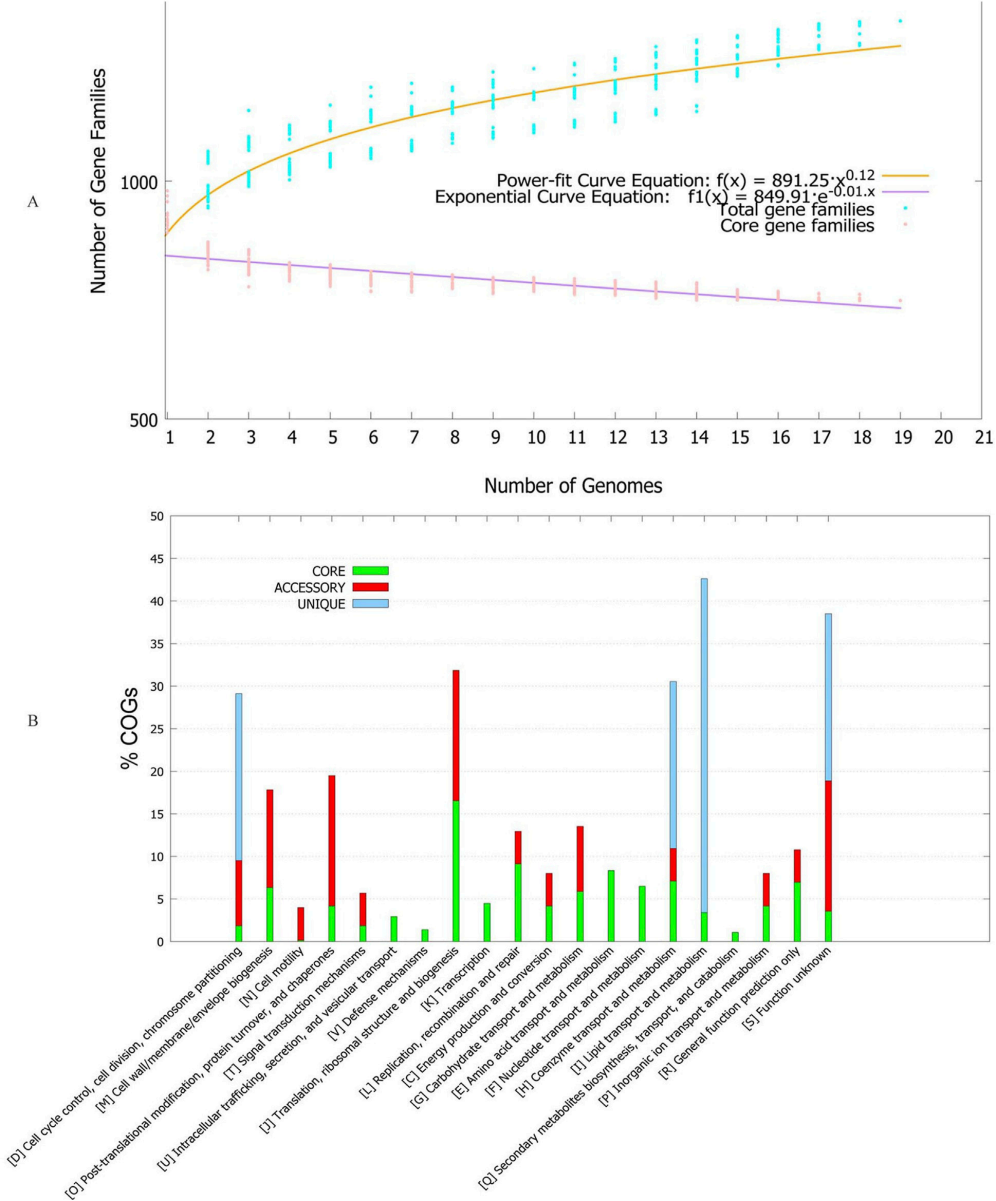
**(A)** Pan-genome accumulation curve of *Tropheryma whipplei* based on analysis of multiple genomes. The curve is modeled using an exponential regression equation, illustrating the growth of the pan-genome as more genomes are added. The x-axis represents the number of genomes included in the analysis, while the y-axis shows the cumulative number of gene families (orthologous groups). The shape of the curve provides insights into whether the species has an open or closed pan-genome, reflecting genomic diversity and gene acquisition potential. **(B)** Functional classification of genes within different genome fractions, expressed as percentages based on Clusters of Orthologous Groups (COG) categories. Genome fractions include the core genome (genes present in all strains), accessory genome (genes present in some but not all strains), and unique genome (strain-specific genes). Each bar represents the relative abundance of genes associated with specific COG functional categories, such as metabolism, cellular processes, information storage, and poorly characterized functions. This distribution reveals how functional roles are partitioned across conserved and variable regions of the genome.

To understand the genetic composition, we conducted pan-genome analysis. The number of core genes was 749. Number of accessory genes ranged between 133-180 (Table 1). The number of gene families generally increased as more strains were included in the analysis, indicating the presence of some strain-specific genes (Figure 1A). Strain Neuro14 had the highest number of unique genes, with 51 unique genes. This indicates a significant divergence from other strains, as these genes are specific to Strain Neuro14 and not found in any other strain. However, this may also mean contaminated genome assembly. It also had largest number of missing genes, meaning these genes may have been lost during evolution or may be specific to certain serovars.

**TABLE 1:** Summary of core, accessory, and unique genes across analyzed *Tropheryma whipplei* strains.

Genome no.	Strain name	No. of core genes	No. of accessory genes	No. of unique genes	No. of exclusively absent genes
1	Art1	749	155	2	2
2	Art29	749	157	4	0
3	Bcu26	749	154	3	2
4	Dig7	749	147	4	1
5	Dig9	749	153	5	0
6	Dig10	749	163	6	1
7	Dig15	749	155	6	0
8	DigADP25	749	167	5	0
9	DigMusc17	749	177	2	1
10	Endo27	749	152	6	0
11	Endo32	749	163	3	0
12	Neuro1	749	154	7	0
13	Neuro14	749	180	51	13
14	Neuro20	749	174	10	0
15	Pneumo30	749	149	10	0
16	Sali28	749	174	9	1
17	Slow2	749	133	10	2
18	TW0827	749	161	47	5
19	Twist	749	193	27	5

The power fit and exponential curve analysis indicate that as the number of genomes increases, the number of core gene families decreases. The negative exponent indicates that the curve is downward-sloping, suggesting a decreasing rate of change in core gene families with each additional genome. The rate of decrease is controlled by the exponent, with a higher value indicating a faster decay. Over time, the curve approaches an asymptote, representing a stabilization or saturation point where the number of core gene families stabilizes. Hence, the power-fit curve suggests a decreasing rate of change in core gene families with each additional genome, while the exponential curve suggests an initial rapid decay followed by stabilization.

Clusters of Orthologous Groups of proteins (COGs) were also studied for each genome fraction. It is interesting to note that unique genes were only present for cell cycle control, cell division, chromosome partition, lipid transport, lipid metabolism, co-enzyme transport, co-enzyme metabolism and unknown function category of proteins (Figure 1B). Cell motility group was only present for accessory genome, while intracellular transport, defense, transcription, amino acid transport, amino acid metabolism, nucleic acid transport, nucleic acid metabolism, secondary metabolite synthesis/transport/metabolism was only present in the core fraction. Phylogenetic analysis based on core and pan-genes revealed a diverse tree structure, with clusters positioned differently depending on gene allocation (Supplementary Figure S1). This variability underscores the dynamic nature of gene presence and absence across strains. Over time, certain strains may lose specific genes present in others, contributing to distortions in the depicted evolutionary relationships within the tree. While core genes represent a conserved set shared among all strains, they may not fully capture the intricate evolutionary history of a species. Conversely, pan-genes provide a more comprehensive view by encompassing genes present in at least one strain within the population. However, pan-genes can also be heavily influenced by horizontally transferred gene events, introducing complexities into the evolutionary interpretation.

### 3.2 Drug target selection and 3D model study

Core proteome data was subjected to CD-HIT analysis, and a total of 749 sequences from the core proteome were obtained. Among these, 422 sequences were found to be common to the CEG, while 423 sequences were common to the DEG, and 409 sequences were found to be common to both CEG and DEG categories. Upon further analysis, 147 sequences were left after subtracting sequences similar to the human proteome. Subsequently, upon subtraction of these sequences from the gut beneficial microbiota proteome, 37 sequences remained. Within these, six were identified as virulence factors, indicating potential roles in pathogenicity. Only 11 were found to be similar to drug targets listed in the DrugBank database proteins (Supplementary Table S1). Among these, *murE* was selected as a drug target screening Ayurvedic products. *murE* is an enzyme that plays a crucial role in the biosynthesis of bacterial cell-wall peptidoglycan. Specifically, it catalyzes the addition of an amino acid to the nucleotide precursor UDP-N-acetylmuramoyl-L-alanyl-D-glutamate (UMAG). This enzymatic reaction is essential for the construction and maintenance of the bacterial cell wall, which provides structural support and protection to the cell. Hence, it could be used as a potential target for antibacterial agents due to its essential role in for bacterial survival.

3D structure of the *murE* gene product (UDP-N-acetylmuramyl-tripeptide synthetase) was obtained from AlphaFold (https://alphafold.com/entry/Q83HJ9;,retrieved 28 February 2024). Most of the structure has a very high pLDDT >90, depicting good quality (Supplementary Figure S2A). It was also evaluated by assess module of Swiss Model and showed 96.45% residues as Ramachandran favored and only 1.18% as outliers (Supplementary Figure S2B). PAE was low (Supplementary Figure S2C). This is a metric used to assess the accuracy of residue alignments in a protein structure prediction. It measures the expected positional error (in Angstroms) between aligned residues in the predicted model and the corresponding residues in the true structure. A lower PAE value indicated a higher accuracy of residue alignment, meaning that the predicted model closely matched the true structure. It is particularly useful for assessing inter-domain accuracy, as it helps identify regions of the protein structure where the alignment may be less reliable.

### 3.3 Virtual screening

**FIGURE 2:**
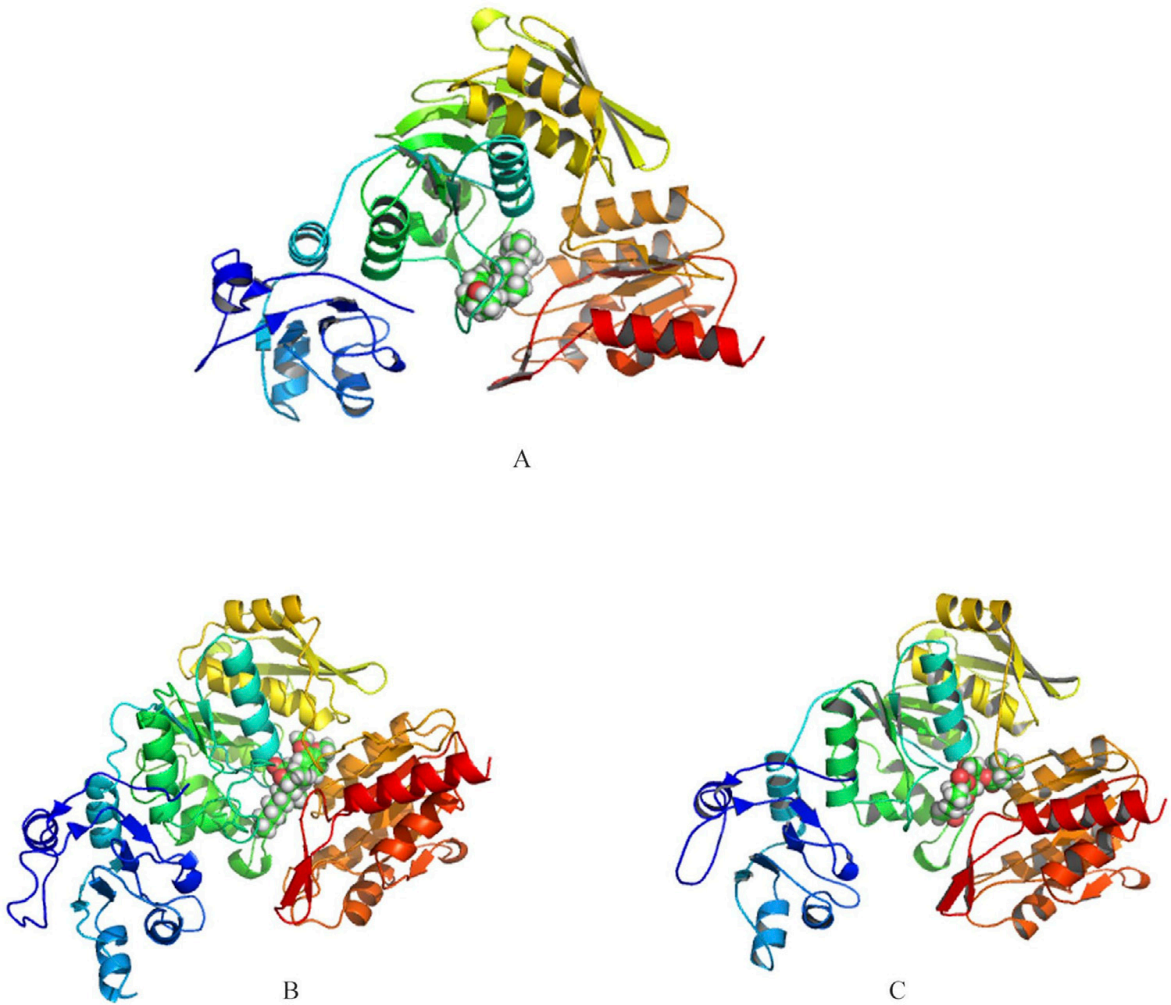
3D representations of the binding interactions between the predicted structure of UDP-N-acetylmuramyl-tripeptide synthetase and selected phytochemicals **(A)** Ergost-5-en-3-ol, (3beta,24xi), **(B)** [6]-Gingerdiol 3-monoacetate and **(C)** Valtrate. Phytochemicals are depicted in space-filling representation, while the protein is shown as a ribbon structure to illustrate the overall fold and ligand-binding orientation.

Autodock revealed top three interactors with lowest energy of −9.1, −8.6 and −7.9 kcal/mol as Ergost-5-en-3-ol, (3beta,24xi) (IUPAC name: (3*S*,8*S*,10*R*,13*R*,17*R*)-17-[(2*R*,5*S*)-5,6-dimethylheptan-2-yl]-10,13-dimethyl-2,3,4,7,8,9,11,12,14,15,16,17-dodecahydro-1*H*-cyclopenta [a]phenanthren-3-ol), [6]-Gingerdiol 3-monoacetate (IUPAC name: [(3*R*,5*S*)-5-hydroxy-1-(4-hydroxy-3-methoxyphenyl)decan-3-yl] acetate) and Valtrate (IUPAC name: [(1*S*,6*S*,7*R*,7*aS*)-4-(acetyloxymethyl)-1-(3-methylbutanoyloxy)spiro [6,7*a*-dihydro-1*H*-cyclopenta [c]pyran-7,2′-oxirane]-6-yl] 3-methylbutanoate) (Figure 2). Ergost-5-en-3-ol, (3beta,24xi), with a molecular weight of 400.7 g/mol, made 19 interactions, with four hydrophobic (Ile152, Ala156, Cys162, Tyr482) and seven hydrophilic (Lys128, Glu158, Arg174, Arg352, Asp479, Asp480, Pro481) residue interactions. [6]-Gingerdiol 3-monoacetate, with a molecular weight of.

338.4 g/mol, made 20 interactions with four hydrophobic (Ala313, Phe314, Met353, Ala374) and seven hydrophilic (Asn126, Lys128, His217, Arg352, Asp367, His368, His370) residues. This compound has been reported from the rhizome of the *Zingiber officinale* (Tao et al., 2008). Its anti-allergic potential has been noted (Chen et al., 2009).

**FIGURE 3:**
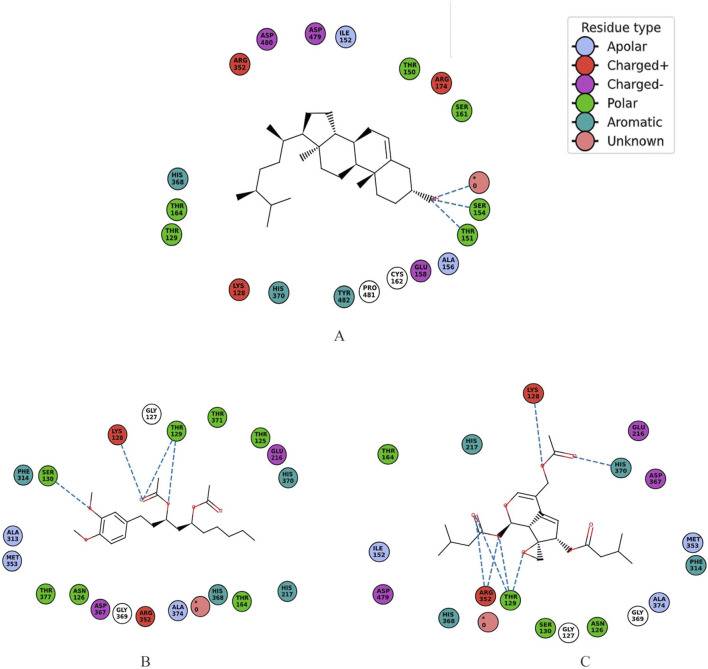
2D schematic representations illustrating the molecular interactions between UDP-N-acetylmuramyl-tripeptide synthetase and three bioactive compounds, detailing specific residue contacts and types of interactions for protein and **(A)** Ergost-5-en-3-ol, (3beta,24xi), **(B)** [6]-Gingerdiol 3-monoacetate and **(C)** Valtrate. The unknown residue shown in pink was not included in the interaction analysis.

Valtrate, with a molecular wight of 422.5 g/mol, made 18 interactions, with four hydrophobic (Ile152, Phe314, Met353, Ala374) and nine hydrophilic (Asn126, Lys128, Glu216, His217, Arg352, Asp367, His368, His370, Asp479) residues (Figure 3). Valtrate belongs to iridoids monoterpenoids and is reported to be isolated from *Valeriana alliariifolia, Valeriana microphylla, Valeriana jatamansi, Valeriana vaginata, Valeriana sorbifolia, V. jatamansi, Valeriana officinalis* and *Valeriana pseudofficinalis* (source LOTUS database, https://lotus.naturalproducts.net/; retrieved on 30 March 2024). It has shown good IC50 against pancreatic cell lines PANC-1 (IC50 = 23.7 ± 1.3 μM), BxPC-3 (IC50 = 20.14 ± 1.2 μM), AsPC-1 (IC50 = 10.67 ± 0.9 μM), Hs 766t (IC50 = 24.12 ± 1.2 μM), and Capan-2 cells (IC50 = 13.45 ± 0.92 μM) for 24 h treatment (Chen et al., 2021). It accomplished anti-pancreatic cancer effect by inhibiting Stat3 signaling. It also exhibits apoptosis of breast cancer cells and their migration (Tian et al., 2020). In Glioblastoma, analysis of PDGFRA protein and downstream mediators have revealed that Valtrate effectively inhibits PDGFRA/MEK/ERK signaling pathways (Liu et al., 2023). It also reduces tumor volume in tumor-bearing nude mice and improves survival outcomes.

The results obtained from the machine learning (ML)-based methods demonstrated a strong correlation with the AutoDock scores, confirming the consistency and reliability of the computational predictions (Table 2). Ergost-5-en-3-ol (3beta,24xi) demonstrated the strongest interaction with the target protein, as indicated by its combined Vinardo score and CNN affinity. [6]-Gingerdiol 3-monoacetate exhibited moderate binding affinity, with a DiffDock confidence score of −2.08, suggesting slightly greater uncertainty in its binding compared to Ergost-5-en-3-ol. However, its Vinardo score and CNN affinity score still indicated a reasonable strength of interaction. Valtrate showed the weakest binding affinity among the three ligands, with a DiffDock confidence score of −1.41, indicating moderate confidence in its binding. The Vinardo score, along with a lower CNN score and the CNN affinity score, suggests a weaker interaction with the target protein compared to the other two ligands.

**TABLE 2:** Comparative binding affinity scores of candidate compounds docked with UDP-N-acetylmuramyl-tripeptide synthetase, using AutoDock, DiffDock, and CNN-based methods.

Ligand	AutoDock score (Kcal/mol)	DiffDock confidence score	Vinardo score	CNN score	CNN affinity (Kcal/mol)
Ergost-5-en-3-ol, (3beta,24xi)	−9.1	−1.88	−6.465	0.403	6.243
[6]-Gingerdiol 3-monoacetate	−8.6	−2.08	−5.538	0.156	4.604
Valtrate	−7.9	−1.41	−4.689	0.089	4.87

### 3.4 Target promiscuity

All the compounds did not indicate promiscuity (Supplementary Figure S3), using both cell based and target based model. This implies that the compounds exhibited selectivity towards their intended targets without significantly interacting with other off-target molecules or pathways. Such specificity is desirable in drug development, as it minimizes the risk of unintended side effects and enhances the therapeutic efficacy of the compounds. The results obtained from Swiss TargetPrediction (Supplementary Figure S4) for the top 15 enzyme classes revealed distinctive patterns of interaction for Ergost-5-en-3-ol, (3beta,24xi), [6]-Gingerdiol 3-monoacetate, and Valtrate. Ergost-5-en-3-ol, (3beta,24xi) exhibited the highest fraction of interaction with cytochrome P450, suggesting a tendency for metabolic interactions (Jumpa-Ngern et al., 2022). Additionally, it demonstrated significant binding affinity towards LXR-alpha and the androgen receptor, indicating potential involvement in lipid/carbohydrate metabolism (Mohammadi and Oshaghi, 2014) and hormonal regulation (Heilmann-Heimbach et al., 2020), respectively. On the other hand, [6]-Gingerdiol 3-monoacetate displayed affinity for kinases, GPCR-A class, and the nuclear receptor family, suggesting a potential role in signaling pathways and cellular regulation. Valtrate exhibited high affinity for proteases, kinases, and the GPCR A class family, suggesting its potential involvement in modulating various cellular signaling pathways and biological processes.

### 3.5 ADMET properties

ADMET properties were compared for the prioritized compounds (Table 3) and comparison highlighted potential differences between the studied compounds. Ergost-5-en-3-ol, (3beta,24xi) had the lowest water solubility, followed by Valtrate and [6]-Gingerdiol 3-monoacetate, potentially impacting its absorption. To improve absorption, cyclodextrins are a viable option. They are widely recognized as safe excipients in pharmaceutical formulations, with approved applications in drug delivery. Unlike some lipid-based or nanocarrier formulations that may pose toxicity concerns, cyclodextrins generally have low immunogenicity and toxicity, making them a preferred choice for improving drug solubility and stability (Nicolaescu et al., 2025). Hence, AI based cyclodextrin complexation was done for this purpose. SBE-β-CD generally performed well in complexation, particularly for [6]-Gingerdiol 3-monoacetate, whereas other cyclodextrins such as DMCD and TMCD showed comparable but slightly weaker binding (Supplementary Table S2). Valtrate and Ergost-5-en-3-ol, (3beta,24xi) had a more consistent pattern across cyclodextrins, with SBE-β-CD and TMCD generally producing the most favorable results. Overall, SBE-β-CD appeared to be the most effective cyclodextrin for improving the solubility of these compounds.

**TABLE 3:** Absorption, Distribution, Metabolism, Excretion, and Toxicity (ADMET) properties of prioritized inhibitors.

Property	Model name	Ergost-5-en-3-ol, (3beta,24xi)	[6]-Gingerdiol 3-monoacetate	Valtrate	Unit
Absorption	Water solubility	−7.068	−3.607	−4.459	Numeric (log mol/L)
	Caco2 permeability	1.223	0.965	0.968	Numeric (log Papp in 10^–6^ cm/s)
	Intestinal absorption (human)	94.543	89.858	98.778	Numeric (% Absorbed)
	Skin Permeability	−2.86	−2.765	−2.838	Numeric (log Kp)
	P-glycoprotein substrate	No	No	No	Categorical (Yes/No)
	P-glycoprotein I inhibitor	Yes	Yes	Yes	Categorical (Yes/No)
	P-glycoprotein II inhibitor	Yes	No	Yes	Categorical (Yes/No)
Distribution	VDss (human)	0.427	0.27	−0.181	Numeric (log L/kg)
	Fraction unbound (human)	0	0.249	0.194	Numeric (Fu)
	BBB permeability	0.774	−0.466	−1.105	Numeric (log BB)
	CNS permeability	−1.758	−2.858	−2.928	Numeric (log PS)
Metabolism	CYP2D6 substrate	No	No	No	Categorical (Yes/No)
	CYP3A4 substrate	Yes	Yes	Yes	Categorical (Yes/No)
	CYP1A2 inhibitior	No	No	No	Categorical (Yes/No)
	CYP2C19 inhibitior	No	Yes	No	Categorical (Yes/No)
	CYP2C9 inhibitior	No	No	No	Categorical (Yes/No)
	CYP2D6 inhibitior	No	No	No	Categorical (Yes/No)
	CYP3A4 inhibitior	No	No	No	Categorical (Yes/No)
Excretion	Total Clearance	0.572	1.353	0.724	Numeric (log mL/min/kg)
	Renal OCT2 substrate	No	No	No	Categorical (Yes/No)
Toxicity	AMES toxicity	No	No	Yes	Categorical (Yes/No)
	Max. tolerated dose (human)	−0.458	0.787	0.178	Numeric (log mg/kg/day)
	hERG I inhibitor	No	No	No	Categorical (Yes/No)
	hERG II inhibitor	Yes	No	No	Categorical (Yes/No)
	Oral Rat Acute Toxicity (LD50)	2.08	2.014	3.201	Numeric (mol/kg)
	Oral Rat Chronic Toxicity (LOAEL)	0.892	2.03	1.38	Numeric (log mg/kg_bw/day)
	Hepatotoxicity	No	No	No	Categorical (Yes/No)
	Skin Sensitisation	No	No	No	Categorical (Yes/No)
	*T.Pyriformis* toxicity	0.631	1.332	0.308	Numeric (log ug/L)
	Minnow toxicity	−1.94	0.87	0.482	Numeric (log mM)

All compounds had relatively low predicted Caco2 permeability (<1.5), suggesting moderate passive diffusion across cell membranes. They also depicted good predicted intestinal absorption (>89%), where it was highest for Valtrate followed by ergost-5-en-3-ol, (3beta,24xi) and then [6]-Gingerdiol 3-monoacetate. Skin permeability appeared to be similar for all. All three were predicted to be P-gp inhibitors, potentially enhancing their own absorption and that of co-administered drugs. Valtrate had the highest predicted volume of distribution (Vdss), indicating wider distribution in the body. Valtrate and [6]-Gingerdiol 3-monoacetate had predicted protein binding, potentially affecting their free drug concentration available for action.

All of the compounds had low predicted blood-brain barrier (BBB) permeability, suggesting limited brain penetration. None was predicted to be substrates for CYP2D6, a common drug metabolizing enzyme and all were predicted to be substrates for CYP3A4, another major drug metabolizing enzyme. This could impact their half-life and potentially lead to interactions with other CYP3A4 substrates. Valtrate had the highest predicted total clearance, suggesting faster elimination from the body. Only Valtrate was predicted to be a potential Ames mutagen, requiring further investigation. All had relatively high predicted oral rat acute toxicity (LD50) values, suggesting low acute toxicity.

### 3.6 PBPK profile

PBPK values of these compounds under different conditions (normal, cirrhosis, and renal impairment) were calculated (Table 4) and compared. Fraction of the dose absorbed (Fa) from the gastrointestinal tract was high (above 96%) for all compounds, suggesting good oral bioavailability for all compounds, regardless of the condition. Fraction of the dose delivered to the systemic circulation (FDp) after escaping first-pass metabolism was very similar to Fa, implying minimal first-pass metabolism for all compounds. Overall fraction of the dose available systemically was also identical to Fa and FDp due to the minimal first-pass effect.

**TABLE 4:** Physiologically-Based Pharmacokinetic (PBPK) values for prioritized compounds. AUC = area under curve.

Endpoint	Fa [%]	FDp [%]	F [%]	Cmax [ug/mL]	Tmax [h]	AUC(0-inf) [ng-h/ml]	AUC(0-t) [ng-h/ml]
Ergost-5-en-3-ol (3beta,24xi) in normal	3.3489	3.3486	3.3483	0.046	24	943.41	943.41
Ergost-5-en-3-ol (3beta,24xi) in cirrhosis	3.5286	3.5282	3.5282	0.0081	2.1424	504.41	66.041
Ergost-5-en-3-ol (3beta,24xi) in renally impaired	3.3726	3.3722	3.3722	0.0107	2.1952	273.99	85.388
[6]-Gingerdiol 3-monoacetate in normal	97.65	97.65	97.65	0.2772	5.2776	22,180	3822.2
[6]-Gingerdiol 3-monoacetate in cirrhosis	97.62	97.62	97.62	0.1937	5.5336	135,000	2929.9
[6]-Gingerdiol 3-monoacetate in renally impaired	96.948	96.948	96.948	0.2958	5.6008	22,550	4198.5
Valtrate in normal	99.976	99.974	99.974	0.5825	2.3444	294,500	5389.5
Valtrate in cirrhosis	99.919	99.917	99.917	0.4641	2.3236	4,613,000	5091
Valtrate in renally impaired	99.982	99.98	99.98	0.5059	2.2768	289,800	4712.6

Ergost-5-en-3-ol showed a significant decrease in maximum concentration (Cmax) of the drug in the blood after administration under cirrhosis and renal impairment compared to normal, suggesting a potential impact of these conditions on its absorption or distribution. [6]-Gingerdiol 3-monoacetate showed some decrease in Cmax under cirrhosis but not under renal impairment. Valtrate had the highest Cmax in all conditions, and the impact of cirrhosis or renal impairment seemed minimal.

The analysis of pharmacokinetic parameters unveiled intriguing trends for Ergost-5-en-3-ol, [6]-Gingerdiol 3-monoacetate, and Valtrate across different physiological conditions. Ergost-5-en-3-ol exhibited a notable decrease in Tmax under cirrhosis and renal impairment, suggesting unexpectedly faster absorption rates. This phenomenon contradicted the anticipated slower absorption due to compromised liver or kidney function, prompting the need for deeper investigation into its mechanisms. Conversely, [6]-Gingerdiol 3-monoacetate showed a modest increase in Tmax across all conditions compared to the normal state, indicating a tendency for slightly delayed absorption. Meanwhile, Valtrate consistently displayed the shortest Tmax under all conditions, implying rapid absorption independent of physiological status. The AUC(0-inf) (ng-h/mL) parameter represents the area under the concentration-time curve from time zero to infinity, providing a measure of total drug exposure. Ergost-5-en-3-ol exhibited a significant decrease in AUC(0-inf) under cirrhosis and renal impairment compared to normal conditions, aligning with the observed reduction in Cmax. Conversely, [6]-Gingerdiol 3-monoacetate displayed a slight decrease in AUC(0-inf) under cirrhosis but not under renal impairment. Valtrate consistently demonstrated the highest overall AUC(0-inf) across all conditions, indicating the greatest systemic exposure. Similarly, the AUC(0-t) (ng-h/mL) parameter, representing the area under the concentration-time curve from time zero to the last measured time point, mirrored the trends observed for AUC(0-inf).

This analysis underscores the potential impact of liver and kidney function on the pharmacokinetics of these compounds. Ergost-5-en-3-ol demonstrates the most pronounced susceptibility to cirrhosis and renal impairment, as evidenced by reduced Cmax and AUC(0-inf) under these conditions. In contrast, [6]-Gingerdiol 3-monoacetate exhibits a moderate impact of cirrhosis, with slight decreases in both Cmax and AUC(0-inf). Valtrate, on the other hand, appears to be minimally affected by cirrhosis or renal impairment, maintaining consistently high Cmax and AUC(0-inf) across all conditions.

### 3.7 MD simulation

**FIGURE 4:**
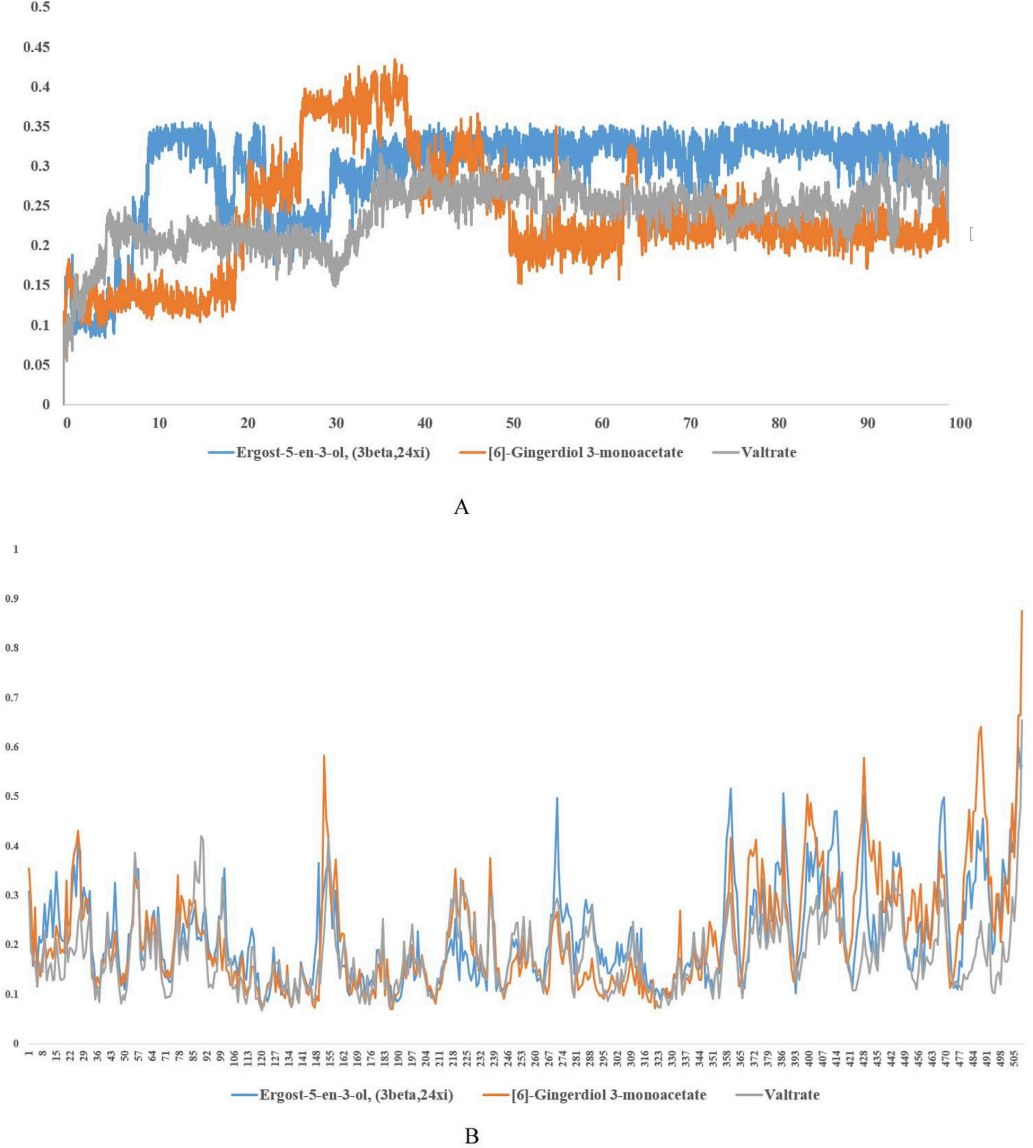
**(A)** Root Mean Square Deviation (RMSD) plot showing the structural stability of UDP-N-acetylmuramyl-tripeptide synthetase in complex with each ligand (Ergost-5-en-3-ol, [6]-Gingerdiol 3-monoacetate, and Valtrate) over a 100 ns molecular dynamics simulation. RMSD values are measured in nanometers (nm) and represent backbone displacement over time, indicating conformational stability and ligand binding persistence. **(B)** Root Mean Square Fluctuation (RMSF) plot of the enzyme residues during the 100 ns simulation for each ligand-bound complex. RMSF values (in nm) reflect the flexibility and local motion of amino acid residues, highlighting regions of structural rigidity or fluctuation upon ligand binding.

**FIGURE 5:**
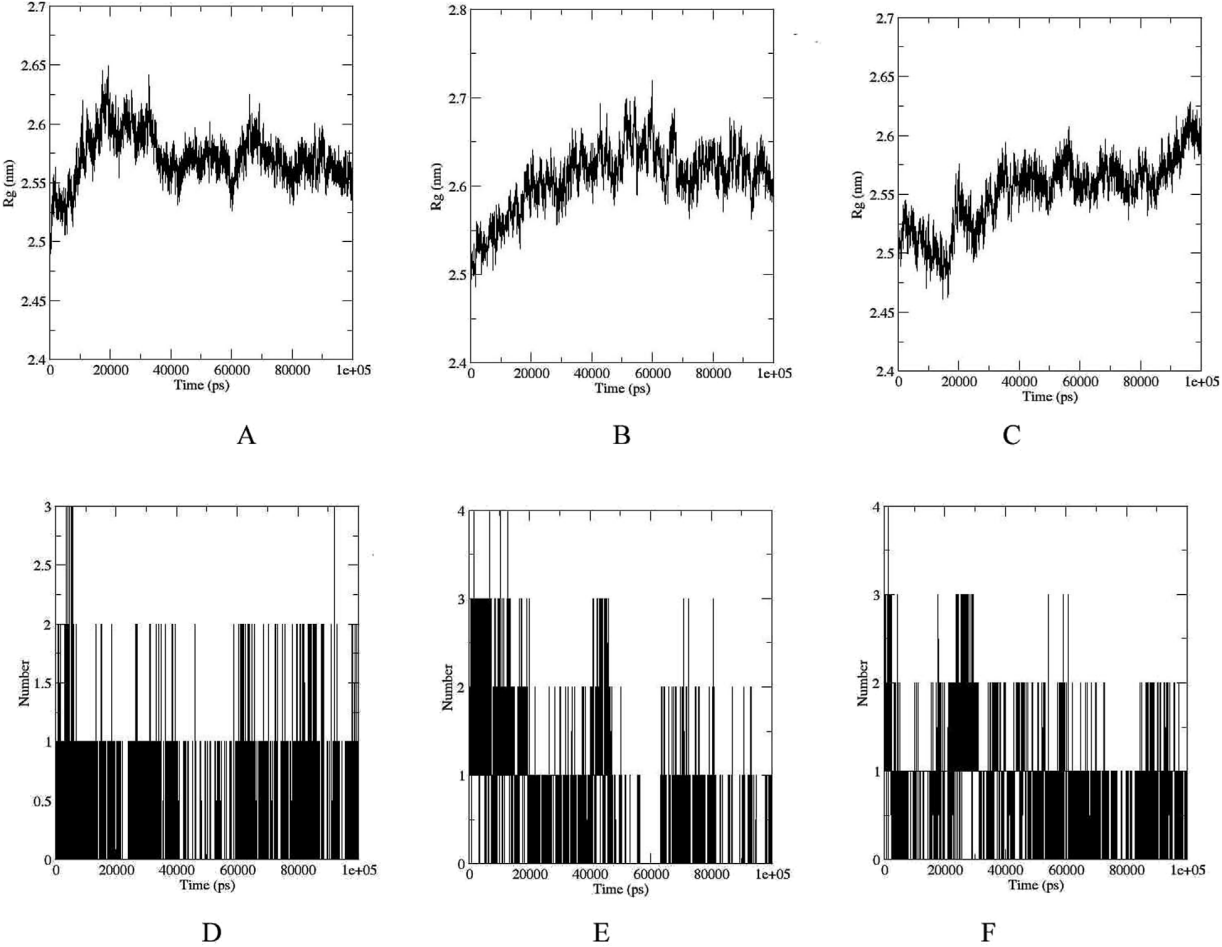
Rg plots of UDP-N-acetylmuramyl-tripeptide synthetase bound with **(A)** Ergost-5-en-3-ol, (3beta,24xi) **(B)** *[6]-Gingerdiol 3-monoacetate* **(C)** Valtrate. Peaks indicate regions of higher mobility, whereas troughs represent structurally stable regions, often corresponding to secondary structure elements or ligand interaction sites. Time evolution of hydrogen bonds formed between the enzyme and each ligand during the simulation: **(D)** Ergost-5-en-3-ol, (3beta,24xi) **(E)** [6]-Gingerdiol 3-monoacetate, and **(F)** Valtrate.

The RMSD, RMSF, and Rg values obtained from molecular dynamics simulations provide valuable insights into the structural stability and flexibility of the studied compounds and their interactions with the UDP-N-acetylmuramyl-tripeptide synthetase protein. The compounds were able to maintain their structural integrity and form stable interactions with the protein. RMSD of UDP-N-acetylmuramyl-tripeptide synthetase and Ergost-5-en-3-ol (3beta,24xi) as well as [6]-Gingerdiol 3-monoacetate was less than or around 0.5 nm (or 5 Å) for the whole simulation period, while it was less than 0.4 nm (4 Å) for Valtrate (Figure 4). For Ergost-5-en-3-ol, (3beta,24xi), it was on average less than 0.35 nm (3.5 Å) and increased upto 4 nm (40 Å) for [6]-Gingerdiol 3-monoacetate, from 20–30 ns but decreased sharply around 50 ns and remained less than 0.25 for rest of the simulation time. For Valtrate, it increased from 0.2–0.25 nm (2–2.5 Å) after 40 ns but stabilized after 50 ns and remained less than 0.3 nm (3 Å) for rest of the time. RMSF was most stable and remained less than 0.25 nm (2.5 Å) for Valtrate, comparatively lower than other compounds. This indicates that it may exhibit less conformational variability and maintain a more rigid structure during interaction with the protein. Rg (Figures 5A–C) also remained less than 0.6 nm (6 Å) for all compounds. Molecules remained relatively compact throughout the simulation, indicating globular or tightly packed 3D structure. Such compactness is often advantageous as it enhances the ability of molecules to passively diffuse through cell membranes. This property is particularly desirable for drugs that need to efficiently reach their target sites within cells but factors like charge and hydrophobicity also play a role in membrane permeability, apart from size and shape.

The analysis of hydrogen bond interactions (Figures 5D–F) revealed that UDP-N-acetylmuramyl-tripeptide synthetase and Ergost-5-en-3-ol, (3beta,24xi) complex did not surpass three hydrogen bonds over the 100 ns simulation period. Moreover, the majority of the time, only two hydrogen bonds were observed. Similarly, for [6]-Gingerdiol 3-monoacetate and Valtrate, the number of hydrogen bonds did not exceed four, with predominantly two bonds observed throughout most of the simulation time. These findings indicate that the compounds may not form extensive or strong hydrogen bonding networks with UDP-N-acetylmuramyl-tripeptide synthetase. Instead, other types of interactions, such as hydrophobic interactions or van der Waals forces, dominate the binding between the compounds and the protein.

PCA on hydrogen bond data (Supplementary Figure S5A) and trajectory RMSD (Supplementary Figure S5B) was also carried out, where hydrogen bond PCA exhibited a linear and scattered distribution along PC1 and PC2, indicative of temporal variability in bonding pattern. This variability reflects intrinsic conformational flexibility rather than structural instability, as no collapse or major disruption was observed. While hydrogen bonds are critical for molecular recognition and structural cohesion, their transient nature during MD simulations is well-documented and does not inherently imply instability. The observed fluctuations in hydrogen bond patterns are consistent with solvent-driven dynamics and conformational flexibility typical of stable biomolecular complexes. Overall stability arises from the interplay of multiple non-covalent interactions, including hydrophobic contacts, van der Waals forces, salt bridges, and entropic effects, which together preserve the structural and energetic resilience of the assemblies. PCA of RMSD data revealed moderate dispersion with overlapping data points across complexes, suggesting structural integrity was largely retained throughout the simulation.

MM/PBSA calculation (Table 5) showed that three compounds demonstrated negative binding free energy (Δ(Complex - Receptor - Ligand)) values, implying favorable interactions. Among them, Valtrate exhibited the most negative Δ value, indicating potentially stronger binding affinity compared to Ergost-5-en-3-ol and [6]-Gingerdiol 3-monoacetate. However, the differences in Δ values between the compounds were relatively small. Overall, the MM/PBSA results suggest that all three compounds have the capacity to bind to the receptor UDP-N-acetylmuramyl-tripeptide synthetase with favorable free energy.

**TABLE 5:** Molecular Mechanics/Poisson–Boltzmann Surface Area (MM/PBSA) values of prioritized compounds over 100 ns. Components include van der Waals, electrostatic, polar solvation, and non-polar solvation contributions.

MM/PBSA energy values	Average MM/PBSA for ergost-5-en-3-ol, (3beta,24xi)	Standard deviation	Average MM/PBSA for [6]-Gingerdiol 3-monoacetate	Standard deviation	Average MM/PBSA for Valtrate	Standard deviation
Complex	−31918.63	319.07	−32018.68	344.44	−31953.99	291.28
Receptor	−32012.72	317.60	−32056.34	340.43	−32019.67	288.81
Ligand	123.48	8.44	61.11	5.96	89.58	6.38
Delta (Complex - Receptor - Ligand)	−29.39	2.43	−23.45	4.18	−23.90	3.23

## 4 Discussion

*Tropheryma whipplei* is a bacterium that appears to be more prevalent than previously believed. Many patients infected with this bacterium exhibit nonspecific symptoms. However, diagnosing this infection poses a significant challenge due to difficulty in the routine cultivation of the bacterium (Dolmans et al., 2017). It is incredibly fastidious and forms cell clumps (Masselot et al., 2003) and is comparatively rare in manifesting itself clinically but can be fatal if untreated (Edouard et al., 2020). There are two stages to the disease caused by it, a prodromal stage characterized by a variety of symptoms linked to persistent nonspecific symptoms including arthritis and arthralgia (Marth et al., 2016). Depending on the region of involvement, the other stage is characterized by weight loss, diarrhea, and other systemic signs (Rocha et al., 2015). For many years, antibiotics were the recommended medication but there have been reports of a significant relapse rate after treatment with penicillin, streptomycin, and tetracycline. Resistance of *T. whipplei* to antibiotic fluoroquinolones has also been reported (Lv et al., 2022). IFN-γ combined with antibiotics or doxycycline combined with hydroxychloroquine has been suggested to overcome resistance, with life-long prescription of doxycycline to prevent relapse (Zhou et al., 2022).

Plant compounds are a good source of drugs due to their diversity and offer a wealth of unique chemical structures with potential therapeutic applications (Lautie et al., 2020). Many widely used medications today have been derived from natural products, such as aspirin (*Salix alba*) (Nica et al., 2020), morphine (*Papaver somniferum*) (Shetge et al., 2020), and digoxin (*Digitalis purpurea*) (Alpert, 2021). Even if a natural product is not a perfect drug, it can serve as a starting point for medicinal chemists to develop more potent and selective derivatives (Berdigaliyev and Aljofan, 2020). Hence, we screened more than 1000 lead-like compounds against UDP-N-acetylmuramyl-tripeptide synthetase of *T. whipplei*, from Ayurvedic medicinal plants. Ayurvedic medicinal plants play a central role in traditional medicine practiced for thousands of years in the Indian subcontinent (Parveen et al., 2020). Indian plants are revered for their therapeutic properties and are utilized to promote health, prevent disease, and treat various ailments (Prasathkumar et al., 2021). Some examples include Ashwagandha (*Withania somnifera*), known for its adaptogenic properties that help the body adapt to stress (Gupta et al., 2021). Turmeric (*Curcuma longa*) is prized for its anti-inflammatory and antioxidant effects (Rolfe et al., 2020; Jantan et al., 2021; Zeng et al., 2021). Tulsi (*Ocimum sanctum*) is revered for its immune-boosting and antimicrobial properties (Rao et al., 2023).

In the current study, we prioritized three compounds (Ergost-5-en-3-ol, [6]-Gingerdiol 3-monoacetate, and Valtrate) by structural docking, against a drug target UDP-N-acetylmuramyl-tripeptide synthetase, from the core genome of *T. whipplei.* These compounds depicted specificity for target as they did not depict a promiscuous nature. They could be used alone or subjected to the exploration of combination therapy approaches, involving synergistic interactions between lead compounds or their combination with existing antibiotics, could enhance treatment outcomes and mitigate the development of resistance (Zhu et al., 2021). While the compounds showed differences in various pharmacokinetic parameters, they generally displayed favorable profiles with regard to ADME. Ergost-5-en-3-ol, (3beta,24xi) exhibited the lowest water solubility among the three compounds, which could potentially hinder its absorption due to reduced dissolution in gastrointestinal fluids. Valtrate and [6]-Gingerdiol 3-monoacetate had comparatively higher water solubility, suggesting better potential for absorption. All compounds showed relatively low predicted Caco2 permeability, indicating moderate passive diffusion across cell membranes. Despite this, they displayed good predicted intestinal absorption (>89%), suggesting efficient absorption in the gastrointestinal tract. All compounds exhibited relatively high predicted oral rat acute toxicity (LD50) values, suggesting low acute toxicity.

Valtrate has been reported to inhibit cancer cell proliferation against various type of tumors (Tian et al., 2020; Chen et al., 2021; Liu et al., 2023) and showed highest predicted total clearance, indicating faster elimination from the body but also depicted AMES toxicity. AMES test is widely accepted assay for evaluating mutagenicity, indicative of potential carcinogenicity in humans (da Silva Dantas et al., 2020). This raises important considerations regarding its therapeutic application and safety profile. This may limit the therapeutic application of Valtrate, especially in long-term treatments or in conditions where prolonged exposure is necessary. Strategies to mitigate genotoxicity, such as structural modifications or combination therapies with protective agents, may be explored to enhance the safety profile of Valtrate.

All compounds exhibited high fractions of the dose absorbed (Fa) from the gastrointestinal tract, exceeding 96%, indicating excellent oral bioavailability across all conditions assessed, including normal, cirrhosis, and renal impairment. Moreover, the fraction of the dose delivered to the systemic circulation (FDp) after escaping first-pass metabolism closely mirrored Fa, indicating minimal first-pass metabolism for all compounds. Consequently, the overall fraction of the dose available systemically was also comparable to Fa and FDp, underscoring the minimal impact of first-pass metabolism on systemic exposure. However, notable differences were observed in the maximum concentration of the drug in the blood (Cmax) among the compounds under different physiological conditions. Ergost-5-en-3-ol exhibited a significant decrease in Cmax under cirrhosis and renal impairment compared to normal, suggesting potential alterations in absorption or distribution kinetics in these conditions. Similarly, [6]-Gingerdiol 3-monoacetate displayed a slight decrease in Cmax under cirrhosis but not under renal impairment. In contrast, Valtrate consistently demonstrated the highest Cmax across all conditions, with minimal impact observed under cirrhosis or renal impairment. These findings suggest differential effects of physiological conditions on the pharmacokinetics of the lead compounds, with Valtrate exhibiting the most robust absorption and distribution profiles. The MD simulation results confirmed the stable binding of the lead compounds to UDP-N-acetylmuramyl-tripeptide synthetase. Examination of intermolecular interactions, including hydrogen bonds and hydrophobic contacts, corroborated the stability of the protein-ligand complex. MM-PBSA analysis further elucidated the binding free energy and contributed to the validation of MD simulation results.

The identification of lead compounds with inhibitory activity against a crucial bacterial enzyme highlights the potential of targeting bacterial cell wall biosynthesis as a strategy for developing novel antibacterial agents (Zhang F. et al., 2022). However, there are certain limitations associated with computer aided drug design studies. While computational virtual screening is a powerful tool for drug discovery, the predictions generated may not always accurately reflect real-world outcomes (Bender and Cortés-Ciriano, 2021; Sadybekov and Katritch, 2023). These *in silico* models often rely on assumptions that may oversimplify biological complexity, such as rigid receptor conformations, lack of solvation dynamics, or incomplete ligand flexibility. To address these limitations, further experimental validation via *in vitro* enzyme inhibition assays and cytotoxicity profiling is critical to confirm the predicted bioactivity, binding affinity, and safety of the lead compounds. *In vivo* studies, including pharmacokinetic and toxicity assessments, are also needed to evaluate therapeutic potential in biological systems. Additionally, the study’s focus on targeting UDP-N-acetylmuramyl-tripeptide synthetase as a therapeutic strategy for Whipple’s disease may overlook other potential targets and treatment modalities, limiting the scope of its findings. A multi-target or systems biology approach may offer a more robust framework for drug discovery in *T. whipplei.* Moreover, the occurrence of AMES toxicity with Valtrate raises concerns about its safety profile and potential mutagenicity, emphasizing the need for further structure–activity relationship investigation and optimization. Lastly, while the findings of this study offer insights into drug discovery for Whipple’s disease, their generalizability to other infectious diseases or bacterial pathogens may be limited, necessitating additional research to assess broader utility.

## 5 Conclusion

The causative agent of Whipple’s disease is *T. whipplei*. To the best of authors knowledge, no natural products have been screened against it so far. In this study, lead like ayurvedic compounds were screened against its protein UDP-N-acetylmuramyl-tripeptide synthetase, essential for cell wall structure. Using a computational virtual screening method, three inhibitors were prioritized. Docking scores and MD simulation confirmed stable interactions of these compounds. Cyclodextrin SBE-β-CD was identified to increase solubility. Despite the occurrence of AMES toxicity with Valtrate, the safety profiles of the lead compounds largely remained favorable, presenting opportunities for further optimization and development. Efforts could be directed towards the optimization of Valtrate to enhance its properties as structure-activity relationship studies and medicinal chemistry approaches can facilitate the design and synthesis of analogs with improved therapeutic profiles. It is proposed to further pursue rigorous *in vitro* and *in vivo* validation to confirm the inhibitory efficacy and safety profiles of the lead compounds.

## Data Availability

Data supporting the research findings is available in the article/Supplementary Material, further inquiries can be directed to the corresponding authors.
