# Supplementary material for: Exploring murE protein inhibitors of Tropheryma whipplei through pharmacoinformatic approaches incorporating solubility-enhancing formulation insights
Source: Front Pharmacol. 2025 Aug 14;16:1630038. doi: 10.3389/fphar.2025.1630038 (PMC12391133; doi:10.3389/fphar.2025.1630038)
Supplement: Supplementary file 1 [file DataSheet1.docx]

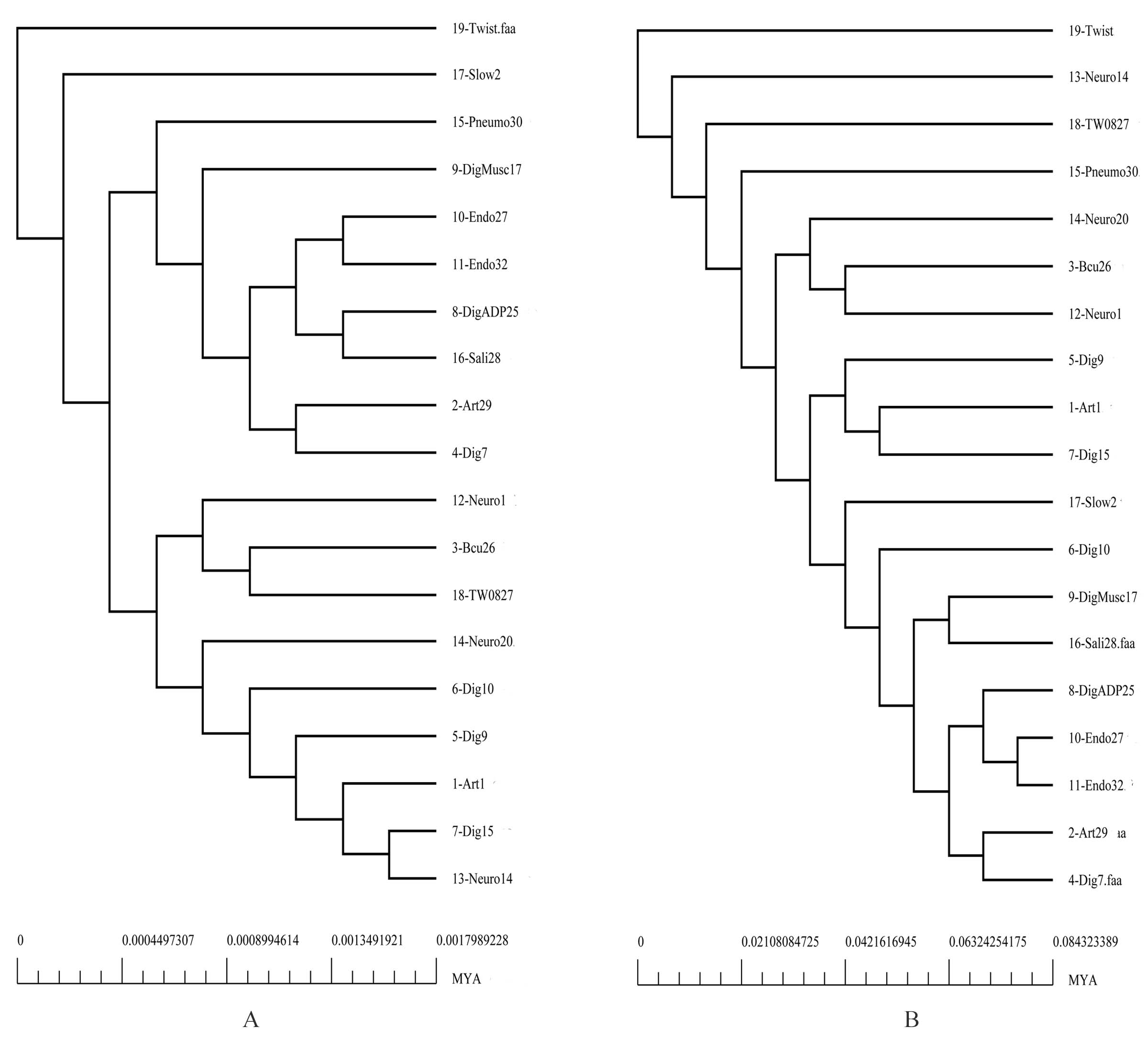


Supplementary Figure 1. (A) Core gene based phylogenetic tree (B) Pan-genome based phylogenetic tree.


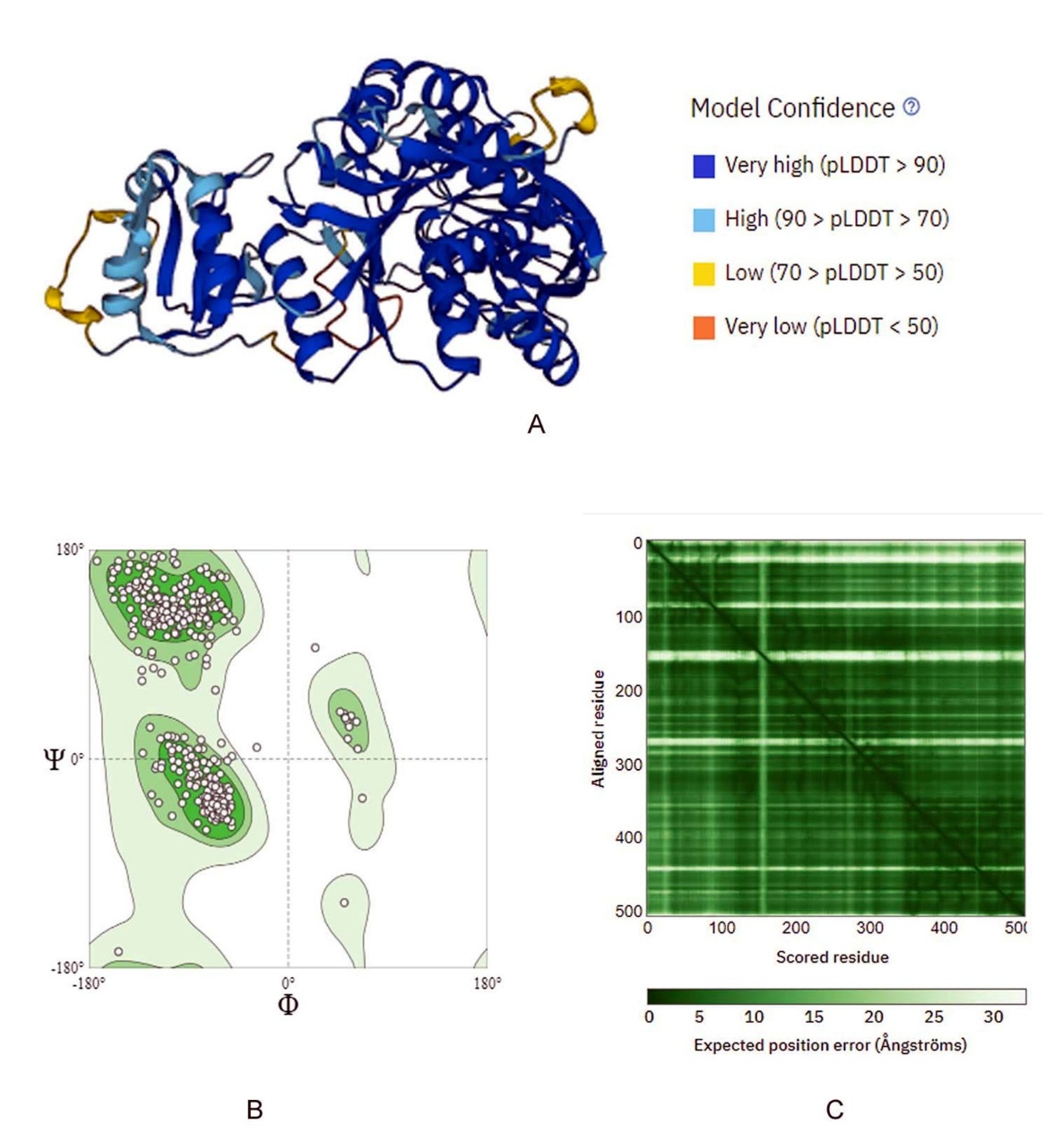


Supplementary Figure 2. (A) Predicted 3D structure of UDP-N-acetylmuramyl-tripeptide synthetase obtained from AlphaFold. The protein model is visualized with secondary structural elements such as α-helices and β-sheets, highlighting domain organization. (B) Ramachandran plot analysis of the predicted structure, illustrating the distribution of backbone dihedral angles (φ and ψ) for all residues. Most residues are located within the favored and allowed regions, indicating good stereochemical quality of the model. (C) Predicted Aligned Error (PAE) plot generated by AlphaFold, showing confidence in the relative positioning of residue pairs across the protein model. Lower PAE values indicate higher confidence in spatial accuracy. The model's PAE profile is also compared against structurally similar proteins in public databases, supporting the structural validity and fold conservation.


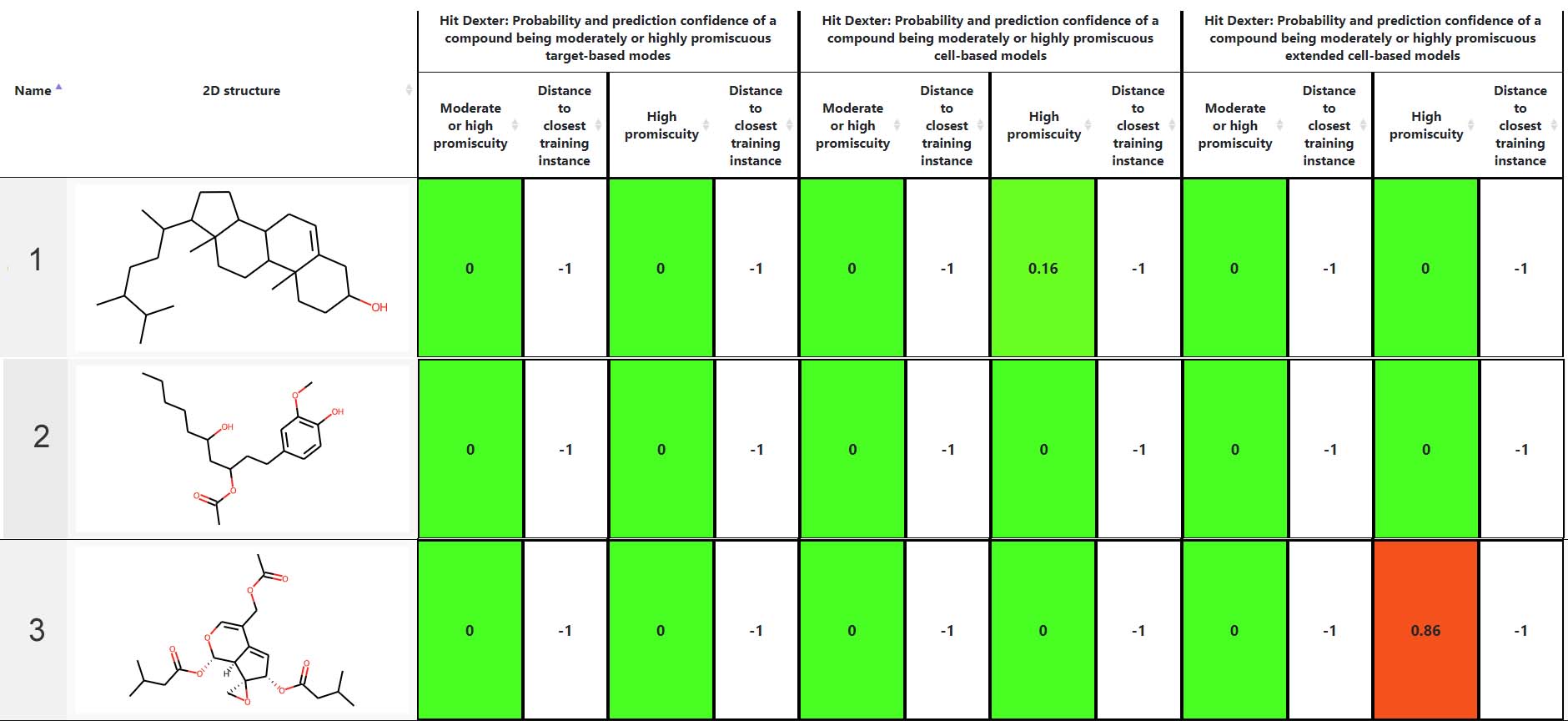


Supplementary Figure 3. 1 refers to Ergost-5-en-3-ol, (3beta,24xi), 2 refers to [6]-Gingerdiol 3-monoacetate and 3 refers to Valtrate.


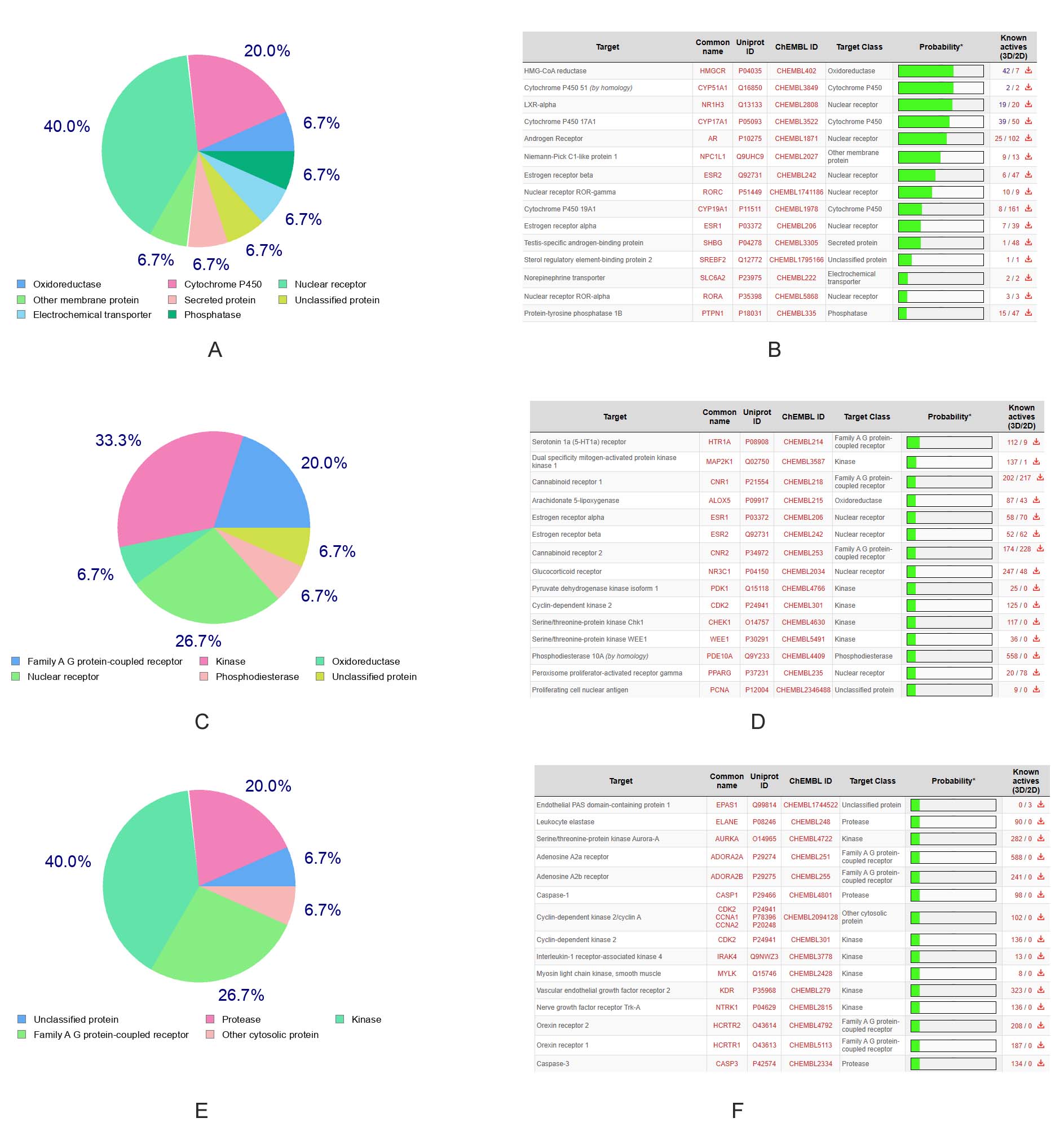


Supplementary Figure 4. (A) Swiss TargetPrediction for top 15 enzyme classes for Ergost-5-en-3-ol, (3beta,24xi) (B) Specific enzyme target for Ergost-5-en-3-ol, (3beta,24xi) among top 15 classes of interactors (D) Swiss TargetPrediction for top 15 enzyme classes for [6]-Gingerdiol 3-monoacetate (E) Specific enzyme target for [6]-Gingerdiol 3-monoacetate among top 15 classes of interactors (F) Swiss TargetPrediction for top 15 enzyme classes for valtrate (G) Specific enzyme target for valtrate among top 15 classes of interactors.


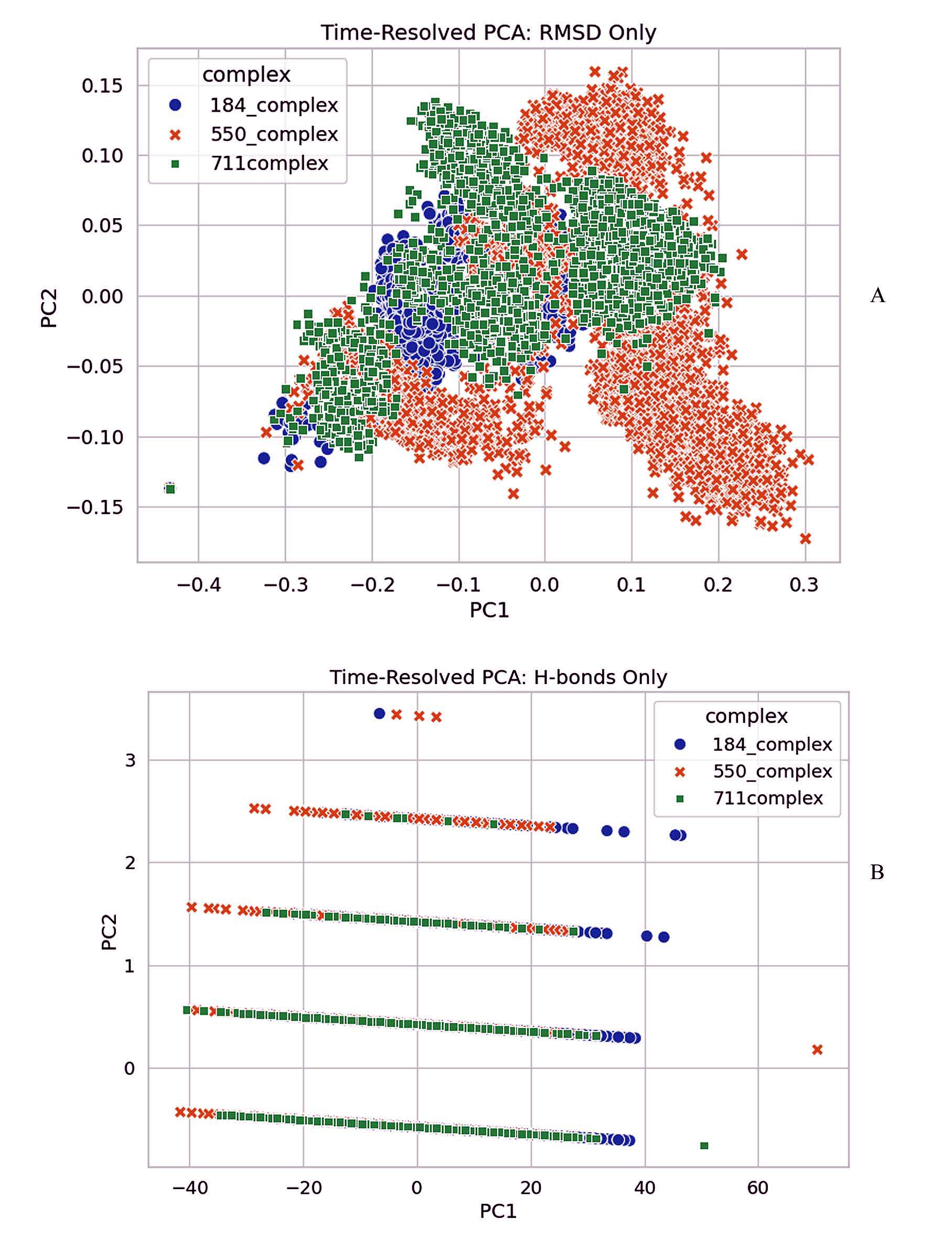


Supplementary Figure 5. (A) Time-resolved PCA based on RMSD data, illustrating the distribution of protein-ligand complexes in the reduced PC1–PC2 space over the 100 ns trajectory. The clustering and overlap of trajectories reflect the conformational stability and similarity among the complexes. The ligands have been shown by their IDs, where 711=Ergost-5-en-3-ol, (3beta,24xi); 550=[6]-Gingerdiol 3-monoacetate; 184=Valtrate (B) Time-resolved PCA of hydrogen bond data, showing the temporal variability in hydrogen bonding patterns. The scattered distribution highlights the dynamic and transient nature of hydrogen bond formation during the simulation. The ligands have been shown by their IDs, where 711=Ergost-5-en-3-ol, (3beta,24xi); 550=[6]-Gingerdiol 3-monoacetate; 184=Valtrate

Supplementary Table 1. Drug target sequences for *T. whipplei.*

| Serial no. | Name | Sequence |
| --- | --- | --- |
|  | 5-methyltetrahydropteroyltriglutamate--homocysteine S-methyltransferase | MLFGTILGYPRLGPRRELRDYLYSYWQGKISEEVLEEKTRLLREETHRRLILLGLSAENY  SIPESFSYYDHVLDAAVHVGAVPSRFSWLLNDGIVDLNAYFTLARGDRSLAPLESLRYFG  SSYHYLVPEIDGNTHFALTSHRVITQFEQAKQAGVNVRPTILGPVSLLMMSRSHGPNAIN  PIERLQDLVPVYSELFEKLYNSGCKFVQVDEPAVTHNTFGIQKSQELLVDAYKFLSSRKL  RPEILVAMSYGNAADQVDALATTGVEAMAFNLIDGAVPFANPSLADKALFGGVIDGRNIW  RGDLAAAYYKLERLRELSGNVVASTSASLFHLPLSLDTEDLGDHLKEWLAFADEKVRQVM  ILARGLEYGVGCIYEHLDSAANSLSDRMTTPGVRVEEVRQALQDLGESDFCRPPREQQQG  CNHASLPILPMVGIGEFSDGTPFVNNDGIPNNNPQAAISFQEDLDILAVQRLGDIDPIQR  FASRMHGFAITHNGWVQSIGPHSVCPPILWGDVSRITPMSSWIEYTQTLTEKSVKPVLPG  PLTLLMSCFVREDQPFQETAQQIAIAIRDDIADLQRSGIRAIQIDEPALEEFLSIYDNAR  YCAPQVFRLATSSAKPEVQIHLHLRPPNLVETIDIINDLDADVISAEIVPYTFGLPAQKC  LMEIRRRIPLCLYFHDANFSRMPSVDECRYLIDTLVRDVDGATLAKQFDDMHIAGFTAGC  AFTAINPRDICVPRADDAGIPSLGSGADHTLERQKATYIPGGIHNLWIGLIYSHSGYNAF  VYDSAYMRDIYIGNELQFVKNMTQAVLLLRKEFTSPQKALSA |
|  | phosphoribosylaminoimidazole carboxylase ATPase subunit | MKDIETITATDAASTEPYAIFLNPRSINRLKTNILTNIPAIPTMPKVAICLYRALFTRQN  ALCMLSIARIASPAQYNLRLMFERLSCSVGVIGGGQLARMMIAPAQALGVDLKVFADTPD  SSAALAATAFGSPENGAAVLEFAQTVDIVTFEHELVPADVLQLLDERGIEMLPRPRALRY  AQDKLALRRYLDEISVNQPAWAEVRSEEDLERFISEHGGCAVVKTGGYDGRGVQVVTLSS  EIDTCRNGGFLAEERIDFVCEVSQLVARSSIGEICVWPLTQTIQENGICVQTITPALGLS  GGNALNQLTTFHDGRTRTPHAPLHSELITKLQDSAKTIALDIARGLNVVGVMAVEMFITQ  DGKLLVNELAMRPHNSGHWSMAASITDQFEQHLRAVLGLPLGATDMSHNCAIMVNIFNSI  SPAQYREVMTHWPDAKLHIYKKQPRPGRKIGHVVFAGEDLQQLAIAAEECRQLLRESHAV |
|  | Mur ligase family protein | MRGCLAIFFGRALLVALRVLRSGGSVLPGVLAEFISPGILIRLIGNSEIIFVTGSNGKTS  TTRMAVSILRAHGHEVFTNSSGANMKYGIIASLLAVPKRTRKGIALLEVDEGHIETLADL  LKPSTALFLNLQVDQLNRFHDPGTVLVKLKNSLAFVKDVLIFNLNEPCFSELLESSLASQ  KLSVYGFSSSSDILNSIPTTGLLPHLGGQFSPPGRIESLCHIEYYSARTAHFSIQGVDRL  QEIQLRTEGVHHAQNAAAAFALCMKILGDEFDPNKASSAVSLSTPAFGRDQVISLAGQPV  RILLMKNPRSMQVNLESIAAGSRVMIGIDGGTPDPSWLYDVNLSAIKRAVVTGSMAWQVA  LALSYAGVVIDLVEPNLFRATNYFLRHSSSDKFQSFYNKKSEVLRKSNFDNNQCVTATHL  DADFNRDQFGAKDDSAMVMILNYEMSMRLLRHYRYLDSSCY |
|  | o-succinylbenzoate synthase | MMDYPSIDTLLESAHIVTLPMKYPFMGLSERQAVLFEGPNSWAEYSPFMGCNDTRMWLAS  AIEYGWGNTNVLSVPNLDLDNTNFASRSKYGYIANPQTSNKDHSDNKRFYTQTKNHREIP  VNAILPDIPASKMSEILGKDLQGCKCVKIKVGKHFQDRDIHRIRAVLDFMGADTMLRLDA  NCKWNVSETLNNLDVLEDNDLLENVSYIEQPCRTTRELITLKQKMDTRGYVTKIAIDESL  RDLLLHWQKNRKWSNNSGITYTTELRETLSACDMLVLKLQPMGGMRAVADLLESIKNNIK  VNVGYTVSSSLETSLGIEMGLALALNLPNITLPVGLATAGLLDADITDDPLLPNKGYITP  RKIEVNKKLLNRYAADKRHVVWWHRRIRAAHRALFQDLSD |
|  | phosphate ABC transporter substrate-binding protein PstS | MDRKLFLVFASVVTLFSVSCFGNSKTSLEGSVSVTGASSQSVAQSVWISEFRKIHPGVTV  NYEPQGSGAGRRAFLSGAVDLAATDVPFADPELTGDFKGCTADGPLQLPVYISPIAIAYN  LPGVSDLKLDASVIAGIFSGAITTWDSPRISALNPGIELPSKKITAVHRSDNSGTTENFS  DYIHTNAPSDWPHKPSTKFPYTYGEAAKGTSGVAQVLKATVGSIGYLDLSLARGMSLATL  KVGSGFSKPSAESATLTVSDSEFPSKNPNSFSVKINRRTEKAGAWPLILVSYVVACVGYK  KGNKDAIFSYLKFILSEKAQGQAAQQAGSAPLPESLSKKLLELVNKIAEKNGA |
|  | ubiquinol-cytochrome c reductase iron-sulfur subunit | MTVSEFKDPGLPPHRQRLTDIDPSAAKRSERIVSVLFFLSALGSVWSIVAYFIFPIESSA  GSRSVHSNNMFFGLGIAFSLLCFGIAAVHWAKTLMLGTEISETRHPIRSSDEDRKQADEI  IKTADSESGFTRRTMIKAALVTALAAFPLPAIVLFRGFAPQEDPVPLLSHTMWKKGIRLV  HDPTGVPIKASDLTVGSIVHVIPDGLLDRHDKLEQKAKAVVLLIRMPLDQLRVSPERKMW  HYKGIVAYSKVCTHLGCPVSLYEHRTHRLLCPCHQSQFDISDEAAVVFGPAARPLPQLPI  TVDSEGYLIAQSDFKEPVGPSFWERSL |
|  | 4-phosphopantoate--beta-alanine ligase | MNLKLASSPHELRTCLAGRAFVLVPTMGALHEGHIWLVDMARRCNLPVVVSIFVNPLQFD  DSLDLDTYPRTLEQDLEKLEGKAFAVYSPSVETMYPNGLDSIRIDPGPIGRILEGAIRPG  FFDGILTIVAKLLLQTAPERVFFSKKDAQQAFLVRRMVRELAFPVRVEVTGFLRDKFSLP  YSSRNRKLGVDAREKAQRLSQGLLSVVNNGPLTVRDCIDKITDLANSIGVDLGYAQILDE  NFCEIASDRMVTRAFHSEACIGLNTPLFLLAARVHGVRVVDNVDLVVV |
|  | aminodeoxychorismate lyase | MNTEYLHVLWFNPDTDCEPRFYDVHHPLIPATSDAINRGVGVFETIGILSGRILNLDEHL  ERMCTSANRLGLRSIEPDRWRSLILQSAKKIADQERAGLRVVYARNSNRSYLAWIAAFAV  RDPDALSKGIKVITLQRGVRSDAGRLYPWLLFGAKTVSYAVNMHALEVAQARGADDAIFL  SEDGLVLEGTTSNLIAYNKGAFITPCPRTMSILPGTTQKRLFMLLEAEGKKTLETSVATE  ALYNSEGVWLTSSVRMITPVVSVDGNRVRFDPGLTDWLNELLARSAV |
|  | pyridoxal 5'-phosphate synthase lyase subunit PdxS | MGLDNLKVGLAQMLKGGVIMDVVTPDQAKIAEDAGAVAVMALEKIPSDIRASGGVSRMSD  PGLIERVMDSVSIPVMAKVRIGHFAEAQILQSLKVDYIDESEVLSVADSSYHIDKRKFTV  PFVCGATNLGEALRRISEGASMIRSKGEAGTGDIAQATKHIRAILSEISALTQAREDELP  ARARELGAPIDLVRETARLGRLPVVLFTAGGIATPADSSLVMQLGSDGVFVGSGIFKSED  PKAYAAAIVQATAQYDDADLLARVSRNLGQAMPGVSNLDVRFSSRGV |
|  | 3-methyl-2-oxobutanoate hydroxymethyltransferase | MSRKPSPTRRTRIHRFHKGSCGRKLVGLTCYDFSTARVLSDCELDFLLVGDSASGVIYGY  ENTGSVCLDEIIYLAAGVVRGAPNSFIIVDLPFGTYEKSDELAVETAIEVIKRTGASAVK  LEGGARMACRISAIVRAGVPVMGHIGFTPQTINALGGYKIQGRDNADLIYLDAQAVEQAG  AFAVVMEMVTEDLAKTITSEIKITTIGVGAGRYTDGQLLVINDLIGLSEKKITFAPRYAS  IDNTVASCVKLWRKDVLEGNFPQKDHIPA |
|  | riboflavin synthase | MFTGLIKNRGVVKDIVRIRGQKSMGAHKNDIAGQITETDSVRKIGVAIPRQHLVQQPKVG  DSVSVNGVCLTVREGHEDVFVFDISQVTADATTINVWDVGQFVNIEMPLTPSDPIGGHIV  QGHVDGVGMIEQIIDNCLRIKIPDSLSLLVVKSGSIAVDGVSLTVVNVSEQLPSTANVSG  NHGKQNCANSWFEINLIPETLERTTLGGLQVGQFVNLETDVLARHVRRFMSLVNSFS |

Supplementary Table 2. ML based validation of prioritized ligands.

| Ligand | Vinardo | DiffDock confidence | CNN Score | CNN Affinity |
| --- | --- | --- | --- | --- |
| Ergost-5-en-3-ol, (3beta,24xi) | -6.465 | -1.88 | 0.403 | 6.243 |
| [6]-Gingerdiol 3-monoacetate | -5.538 | -2.08 | 0.156 | 4.604 |
| Valtrate | -4.689 | -1.41 | 0.089 | 4.87 |

Supplementary Table 3. Cyclodextrin complexation free energy (ΔG) for priority ligands.

| Formulation | DMCD | γ-CD | α-CD | β-CD | TMCD | Hp-β-CD | RMCD | SBE-β-CD |
| --- | --- | --- | --- | --- | --- | --- | --- | --- |
| Ergost-5-en-3-ol, (3beta,24xi) | -13.947 | -13.730 | -12.106 | -13.043 | -13.945 | -13.356 | -13.769 | -13.986 |
| [6]-Gingerdiol 3-monoacetate | -18.174 | -15.709 | -14.692 | -17.265 | -18.168 | -17.951 | -18.146 | -18.929 |
| Valtrate | -16.673 | -14.307 | -13.422 | -15.944 | -16.621 | -16.355 | -16.698 | -16.955 |
